# Establishing the Bases for Introducing the Unexplored Portuguese Common Bean Germplasm into the Breeding World

**DOI:** 10.3389/fpls.2017.01296

**Published:** 2017-07-26

**Authors:** Susana T. Leitão, Marco Dinis, Maria M. Veloso, Zlatko Šatović, Maria C. Vaz Patto

**Affiliations:** ^1^Instituto de Tecnologia Química e Biológica António Xavier, Universidade Nova de Lisboa Oeiras, Portugal; ^2^Unidade de Investigação de Biotecnologia e Recursos Genéticos, Instituto Nacional de Investigação Agrária e Veterinária Oeiras, Portugal; ^3^Faculty of Agriculture, University of Zagreb Zagreb, Croatia; ^4^Centre of Excellence for Biodiversity and Molecular Plant Breeding Zagreb, Croatia

**Keywords:** *Phaseolus vulgaris* L., Portugal, genetic and morphological diversity, admixture, core collection

## Abstract

Common bean (*Phaseolus vulgaris* L.) is among the most important grain legumes for human consumption worldwide. Portugal has a potentially promising common bean germplasm, resulting from more than five centuries of natural adaptation and farmers' selection. Nevertheless, limited characterization of this resource hampers its exploitation by breeding programs. To support a more efficient conservation of the national bean germplasm and promote its use in crop improvement, we performed, for the first time, a simultaneous molecular marker (21 microsatellites and a DNA marker for phaseolin-type diversity analysis) and seed and plant morphological characterization (14 traits) of 175 accessions from Portuguese mainland and islands traditional bean-growing regions. A total of 188 different alleles were identified and an average pairwise Cavalli-Sforza and Edwards' chord genetic distance of 0.193 was estimated among accessions. To relate the Portuguese germplasm with the global common bean diversity, 17 wild relatives and representative accessions from the Andean and Mesoamerican gene pools were evaluated at the molecular level. No correlation was detected between the variability found and the geographic origin of accessions. Structure analysis divided the collection into three main clusters. Most of the Portuguese accessions grouped with the race representatives and wild relatives from the Andean region. One third of the national germplasm had admixed genetic origin and might represent putative hybrids among gene pools from the two original centers of domestication in the Andes and Mesoamerica. The molecular marker-based classification was largely congruent with the three most frequent phaseolin haplotype patterns observed in the accessions analyzed. Seed and plant morphological characterization of 150 Portuguese common bean accessions revealed a clear separation among genetic structure and phaseolin haplotype groups of accessions, with seed size and shape and the number of locules per pod the most discriminant traits. Additionally, we used molecular and morphological data to develop a series of smaller core collections that, by maximizing the genetic and morphological diversity of the original collection, represents the Portuguese common bean germplasm with minimum repetitiveness. A core collection with 37 accessions contained 100% of the genetic variation found in the entire collection. This core collection is appropriate for a more detailed characterization and should be explored, as a priority, in national and international common bean breeding efforts. Furthermore, the identified intermediate accessions (with admixed genetic origin) may have novel genetic combinations useful in future bean breeding.

## Introduction

Common bean (*Phaseolus vulgaris* L., 2n = 2x = 22), from now on designated as bean, is a predominantly self-pollinated herbaceous annual plant, grown worldwide for its edible green pods and dry seeds. As a member of the Fabaceae (legume) family, it has an important role in sustainable agriculture due to its capability for fixing atmospheric nitrogen, allowing for the reduction in fertilizer use. Bean has recognized benefits to human health and nutrition, with its high protein content, dietary fiber, and essential vitamins and minerals (Câmara et al., 2013; Petry et al., 2015). This species is the most consumed grain legume in human diets, with 30 million ha harvested and a global production estimated at 25 million tons in 2014, almost double that of chickpeas and dry peas, the next most consumed grain legumes (FAOSTAT, 2014^1^). As a healthy and inexpensive alternative to animal protein, bean is vital in African (e.g., Burundi and Rwanda) developing countries' nutrition. In Latin America (e.g., Nicaragua, Brazil, and Mexico), bean is an important part of the staple diet (Petry et al., 2015).

In Portugal, bean represents about 75% of the grain legumes consumed by humans but, although there are many bean landraces still in cultivation, especially in the northern and central regions of Portugal, national production covers only 9.4% of the country's demand (“Estatísticas Agrícolas 2015,” www.ine.pt).

Wild bean originated in the Mesoamerican region. It is estimated that 111,000 years ago, a common ancestor diverged into two geographic gene pools, Mesoamerican and the Andean, which were domesticated independently (Bitocchi et al., 2013) giving rise to two gene pools: the Mesoamerican and the Andean. These genetically differentiated pools contain landraces with different seed and leaf sizes, growth habits and seed coat colors and patterns. Based on morphological traits and breeding behavior, different races were defined by Singh et al. (1991), namely *Mesoamerica, Jalisco*, and *Durango* (within the Mesoamerican pool), and *Nueva Granada, Peru* and *Chile* (within the Andean pool). In general, the Mesoamerican germplasm have small (<25 g 100-seed weight^−1^) or medium (25–40 g 100-seed weight^−1^) seed sizes and S or B phaseolin types (the major seed-storage proteins of beans). In contrast, in the Andean germplasm, seeds are predominantly large (>40 g 100-seed weight^−1^) and have T, C, H, or A phaseolin types (Gepts et al., 1986; Singh et al., 1991). Determinacy, characterized by stems ending with a terminal inflorescence, is almost exclusively distributed in the Andean gene pool, probably because in this region bean was domesticated prior to maize and thus grew without a physical support (Koinange et al., 1996; Kwak et al., 2012). In contrast, in the Mesoamerican region, maize and bean have been cultivated together in traditional agriculture and indeterminate types are more common, since bean could use maize plants as supports (Koinange et al., 1996). In addition to these traits, both gene pools present a diversity of seeds colors and shapes, and leaf and bracteole shapes and sizes.

It is known that bean dissemination from the Americas into Europe started during the Columbian Exchange in the sixteenth and seventeenth centuries (Gepts and Bliss, 1988). Portuguese and Spanish sailors and traders first introduced bean seeds to Europe from Mesoamerica, given that Columbus arrived in Central America in 1492 and Cortés reached Mexico in 1518. The introduction of the Andean bean germplasm occurred later, after 1528, with Pizarro's return from Peru (Papa et al., 2006). During the last five centuries, extensive gene flow between these pools, with the development of many intermediate types, has been suggested by several authors, in the Iberian Peninsula as well as in the rest of Europe (Santalla et al., 2002; Logozzo et al., 2007; Gioia et al., 2013). Indeed, about 44% of the European bean germplasm resulted from hybridization between the Andean and Mesoamerican gene pools, a percentage that is four to ten times larger than those observed in the Americas (4.3–12.3%) (Angioi et al., 2010; Burle et al., 2010). The Iberian Peninsula is considered a secondary center of bean genetic diversity (Santalla et al., 2002), since new forms, better adapted to prevailing conditions in this geographic area, emerged from the initial recombination events between the Mesoamerican and Andean gene pools. These intermediate accessions may contain new combinations of genes of numerous agronomic, technological processing, nutritional and organoleptic bean traits. As an example of these interesting trait combinations, broad-based disease resistance may be achieved in bean by combining resistance genes from both gene pools, since the co-evolution of host and pathogen within gene pools is frequent. As has previously been described, resistance genes from Mesoamerican origin are very effective when transferred to beans of Andean background and vice-versa (Guzman et al., 1995; Geffroy et al., 1999; Miklas et al., 2006).

In Portugal, a great diversity of bean landraces has been cultivated for generations and is still maintained in small fields for farmers' personal consumption and to sell in local markets (Vaz Patto et al., 2007; Leitão et al., 2013). Nevertheless, yield instability, especially due to diseases, pests and drought susceptibility make bean less attractive for extensive farming, particularly under Mediterranean conditions in which heat waves and periods of drought are becoming more frequent. Consequently, in Portugal but also throughout the rest of Europe, there is no high-quality raw bean material production in significant amounts and the processing industries rely completely on foreign materials.

Germplasm collections, hosting traditional landraces, have an important value in biodiversity preservation, development of genetic studies and progress in plant breeding. However, these plant resources must be characterized for morphological, quality and agronomic traits as well as for their genetic diversity, to be effectively conserved and used for breeding. Portugal has considerable bean germplasm resources, which due to their highly diverse and putative intermediate nature, represent important sources of interesting plant traits combinations not yet explored in breeding programs. However, the few studies performed so far to characterize these resources, namely by assessing their genetic variability, were limited to a small number of accessions, collected from a few geographic regions, mainly northern mainland Portugal (Rodiño et al., 2001; Santalla et al., 2002; Martins et al., 2006; Coelho et al., 2009) and Madeira Island (Freitas et al., 2010). Nevertheless, these studies showed that the few Portuguese accessions analyzed were closer to the Andean gene pool and contained significant genetic variability useful for plant improvement in traits such as plant height, seed color and shape, and protein content.

In order to support a more efficient conservation of the national bean germplasm, and to enhance its use in crop improvement, we need to enrich our current understanding of the relationships among accessions and the underlying patterns of diversity through the analysis of a more representative collection. To achieve that, in the present study, we characterized the genetic diversity and structure of an enlarged bean collection, with 175 accessions, originating in all traditional Portuguese bean-growing geographic regions, using microsatellite markers. The genetic diversity of this collection was compared with representative accessions from the Andean and Mesoamerican gene pools, in order to position the Portuguese germplasm in the worldwide diversity of bean. Moreover, we improved the limited available characterization on seed and plant morphological and agronomical traits of the Portuguese bean germplasm. Based on the molecular and morphological data collected, we developed a series of smaller core collections of bean accessions. By capturing, with minimum repetitiveness, the maximum allelic diversity, these collections are more suitable for further detailed characterization of specific traits, and can help to achieve an increased efficiency in the utilization of Portuguese sources in the improvement of this important legume crop.

## Materials and methods

### Plant material

The 175 Portuguese bean accessions used in the genetic diversity study were selected based on the national geographical distribution of their collecting sites. One hundred and seven accessions were collected by visiting small farms in a field expedition that took place in 2005 (Vaz Patto et al., 2007), while the remaining accessions were selected from the collection held at the Research Unit of Biotechnology and Genetic Resources, INIAV, Oeiras, Portugal. Based on the available seed, a subset of 150 accessions was selected for morphological evaluation. From the total evaluated accessions, 166 were from mainland Portugal and 9 from the autonomous regions (Azores and Madeira archipelagoes). In more detail, 61 accessions (34.8%) were from the northern interior of mainland Portugal, 11 (6.3%) from the north coast, 68 (38.8%) from the central north, 12 (6.8%) from the central south, 14 (8.0%) from the south, 1 (0.57%) from S. Miguel Island (in the Azores) and 8 (4.6%) from Madeira Island. Maps indicating the collecting sites of the Portuguese accessions, as well as the national climatic data and soil main characteristics are available in Supplementary Figures 1–4. In addition, 17 bean accessions were selected from the CIAT seed bank collection, 11 based on their gene pool and eco-geographic race and 6 wild relatives. From those 17 accessions, 5 were representatives of the Mesoamerican gene pool (2 from race *Mesoamerica*, 2 from race *Guatemala* and 1 from race *Jalisco*), 6 were representatives of the Andean gene pool (2 from race *Peru*, 2 from race *Chile* and 2 from race *Nueva Granada*) and the 6 wild accessions were from Argentina, Bolivia, Colombia, Guatemala, Mexico and Peru. A complete list of the accessions studied, along with their “passport” information, is available in Supplementary Table 1.

### DNA isolation

Ten seeds randomly selected from each of the 192 accessions (175 Portuguese plus 17 from the CIAT collection) were sown. Individual young leaves were collected, then immediately frozen in liquid nitrogen and stored at −80°C until DNA isolation. DNA was isolated using a modified CTAB protocol developed by Torres et al. (1993). DNA quality was evaluated on 0.8% agarose gels (Lonza, Rockland, USA) with SYBRSafe (Invitrogen, Eugene, USA) staining and visualized using a GEL-DOC1000 System (Bio-Rad, Hercules, USA), followed by quantification in a Nanodrop spectrophotometer (Thermo Scientific, Passau, Germany). DNA was diluted in TE buffer to 10 ng/μL for subsequent experiments. In total, DNA from 1826 individuals was isolated (9.5 individuals per accession on average with a minimum of 6 individuals per accession).

### Fluorescent microsatellite analysis

The selection of the 21 microsatellite markers (simple sequence repeats, SSR) used was made based on their uniform distribution and coverage of the 11 chromosomes of the bean genome (Yu et al., 2000; Gaitan-Solis et al., 2002; Blair et al., 2003). Two types of SSRs were included: for coding (gene-derived markers) and non-coding (SSRs derived from non-coding genomic DNA) regions (Supplementary Table 2).

The method for fluorescent labeling of PCR fragments developed by Schuelke (2000) was used. Accordingly, a M13 tail was added to the 5″-end of the forward primers, which allows the annealing of the universal M13(-21) primer labeled with IRDye fluorescence and the visualization of the amplified fragments, resolved in a 6.5% polyacrylamide gel (KBPlus Gel Matrix, LI-COR), using a LI-COR 4300 DNA Analyzer (Lincoln, NE, USA). PCR reactions were conducted in a total volume of 10 μL containing 10 ng of template DNA, 0.04 μM of M13(-21) tagged forward primer, 0.16 μM of IRD700 or IRD 800 M13(-21) and 0.16 μM of reverse primer, 0.2 mM of each dNTP, 1.5 mM of MgCl2, and 0.2 units of Taq DNA polymerase (Promega, Madison, USA). The amplification reactions consisted of a denaturing step of 5 min at 94°C, followed by 30 cycles of 30 s at 94°C, 45 s at 56°C, 45 s at 72°C, and 8 cycles of 30 s at 94°C, 45 s at 53°C, 45 s at 72°C. The reactions were terminated at 72°C for 10 min. After the amplification, 1 uL from each reaction product was mixed with 25 μL of formamide-loading buffer (98% formamide, 10 mM EDTA pH = 8.0 and 0.1% Bromo Phenol Blue). The total mixture was carefully vortexed, heated for 5 min at 95°C in a denaturation hotblock and then quickly cooled on ice. 0.5 to 0.7 μl of each sample was loaded on the 6.5% denaturing polyacrylamide gel.

Allele sizes (in base pairs) of PCR products were estimated using SAGA^GT^ software from LI-COR and data compiled in a matrix for further analysis.

### Phaseolin type analysis

A DNA marker for phaseolin-type diversity analysis was used (Kami et al., 1995), which specifically amplifies a region surrounding the 15-bp tandem direct repeat of the phaseolin gene family. The method for fluorescent labeling of PCR fragments developed by Schuelke (2000) was used and the PCR conditions were the same described above for the SSR markers.

### Molecular data analysis

To verify the usefulness of the molecular markers selected to assess the genetic diversity of the accessions analyzed, the Polymorphism Information Content (PIC) of each microsatellite marker was calculated by PowerMarker v3.23 software (Liu and Muse, 2005). GENEPOP v4.0 (Rousset, 2008) was used to calculate the average number of alleles per locus (*N*_*av*_) and the observed (*H*_*O*_) and expected heterozygosity (*H*_*E*_, or gene diversity) of each accession. The effective number of alleles (*N*_*e*_) was estimated using GenAlEx 6.5 (Peakall and Smouse, 2006). The allelic richness (*N*_*ar*_), as the measure of the number of alleles per locus independent of sample size was calculated using FSTAT v2.9.3.2 program package (Goudet, 1995, 2002), while the number of private alleles per accession (alleles present in a subgroup of a broader collection of accessions that are indicative of gene pool differentiation) was assessed using MICROSAT software (Minch et al., 1997). Private allelic richness (*N*_*par*_) within each accession was estimated after controlling for differences in sample size using the rarefaction method (Kalinowski, 2004) implemented in the program HP-Rare (Kalinowski, 2005).

To test the significance of the differences in *N*_*ar*_, *H*_*O*_, and *H*_*E*_ between two groups of accessions (Portuguese vs. Andean and Mesoamerican race representatives and wild relatives) as well as among accessions originating from different regions in Portugal, Kruskal-Wallis (among all groups) and Wilcoxon (between all possible pairs of groups) non-parametric tests were performed using SAS/STAT® Software (2004). Furthermore, the differences in *N*_*ar*_, *H*_*O*_, and *H*_*E*_ between two groups (Portuguese vs. Andean and Mesoamerican race representatives and wild relatives) were tested on individual level across microsatellite markers by repeated measures analysis of variance using PROC GLM in SAS/STAT® Software (2004).

To check for a possible correlation between the geographic distance and the genetic differentiation of the accessions, isolation by distance (IBD) among Portuguese accessions was tested using the method of Rousset (1997). For this analysis, we only considered the 134 accessions from which the GPS coordinates of their local of origin were available and excluded the insular accessions from Madeira and the Azores. A Mantel test (106 permutations of accession locations among all locations) on the matrix of pairwise FST/(1-FST) ratios and that of the natural logarithm of geographical distances (in km) between pairs of accessions was performed using NTSYS-pc v2.02 (Rohlf, 1997).

To test for the existence of genetic structure in this collection, an analysis of molecular variance (AMOVA) was performed, based on SSR markers, using ARLEQUIN v3.0 (Excoffier et al., 2005). We tested four partitions of variance: (A) among and within all bean accessions; (B) among groups of accessions (Portuguese vs. gene pools representatives and wild relatives), among accessions within groups and within accessions, as well as among and within accessions separately for Portuguese (C) accessions and gene pools representatives and wild relatives (D). Variance components were tested statistically by non-parametric randomization tests using 10,000 permutations.

Pairwise Cavalli-Sforza and Edwards' chord distances (Cavalli-Sforza and Edwards, 1967) were calculated and cluster analysis was performed using Neighbor-joining algorithm with 1,000 bootstraps (Felsenstein, 1985) over microsatellite loci as implemented in SEQBOOT, GENDIST, NEIGHBOR, and CONSENSE programs of the PHYLIP v3.6b software package (Felsenstein, 2004). In addition, the genetic distance matrix obtained was used to construct a dendrogram through the Neighbor-joining algorithm.

A model-based clustering method was applied on multilocus microsatellite data to infer genetic structure and define the number of clusters in the dataset using the software STRUCTURE v2.3.3 (Pritchard et al., 2000). Ten runs per each cluster ranging from 1 to 11 were carried out on the Isabella computer cluster at the University of Zagreb, University Computing Centre (SRCE). Each run consisted of a burn-in period of 200,000 steps followed by 10^6^ MCMC (Monte Carlo Markov Chain) replicates assuming admixture model and correlated allele frequencies. No prior information was used to define the clusters. The choice of the most likely number of clusters (K) was carried out by comparing the average estimates of the likelihood of the data, ln[Pr(X|K)], for each value of K, as well as by calculating an *ad hoc* statistic ΔK, based on the rate of change in the log probability of data between successive K values as described by Evanno et al. (2005). The software program STRUCTURE HARVESTER v0.6.92 was used to process the STRUCTURE results files. The runs with the maximum likelihood were chosen and by averaging the estimated membership coefficients of the individuals, the proportion of ancestry of each accession in each of the clusters was calculated. The accessions were assigned to a particular cluster if at least 75% of their genome was estimated to belong to that cluster while those accessions with value <75% for all the clusters were considered of admixed origin.

### Assessment of phaseolin types and relationship with structure memberships

Phaseolin type of each accession was assessed based on the pattern of the fragments amplified with the phaseolin molecular marker (Kami et al., 1995). The phaseolin patterns obtained in the Portuguese accessions were compared with the ones from CIAT accessions (Andean and Mesoamerican race representatives and wild relatives), from which the characteristic phaseolin type is known. The phaseolin pattern of all the accessions was compared to the clustering obtained after structure analysis, in order to verify the correspondence between the phaseolin types and the structure membership obtained. Based on this correspondence, accessions were categorized as true-types or as offtypes according to the following four designations: (1) True-type: unique type of phaseolin within all individuals of each accession, percentage of cluster membership (Q in structure analysis) higher than 75% and phaseolin type matching the cluster membership; (2) Offtype - Composite: Q < 75% at accession level, but with less than 50% of their individuals with Q < 75%; (3) Offtype - Hybrid: Q < 75% at the accession level, but with at least 50% of the individuals with Q < 75%; (4) Offtype - non-corresponding: Q > 75% at accession level, but with more than one phaseolin type and/or no correspondence between clusters and phaseolin types.

Since accessions classified as offtype had an admixture nature and could be considered putative hybrids, the subsequent statistical tests performed, to compare molecular and morphological diversity among groups of accessions, were done only for true-type accessions. To test the significance of the differences in *N*_*ar*_, *H*_*O*_, and *H*_*E*_ among true-type groups of accessions, Kruskal-Wallis (among all groups) and Wilcoxon (between all possible pairs of groups) non-parametric tests were performed using SAS/STAT® Software (2004).

### Phenotypic data analysis

A total of 150 Portuguese bean accessions were grown during 2014 in a farmer's field at Cabrela (near Sintra, Portugal) in 3.0 m-rows spaced 50 cm using traditional farmers' production management. The soil was a haplic luvisol, and during the vegetative cycle (May to August/September) the temperature ranged from 18°C to 28°C (mean values of minimum and maximum temperatures). Accessions revealing a climbing habit were stacked to permit a full development of the plants.

Data on morphological and agronomic traits were collected from seeds and plants, following the descriptors for *Phaseolus vulgaris* L. (IBPGR, 1982). Ten plants per accession were used to measure the number of seeds per pod, the number of locules per pod (considering only the longest pod) and the plant growth habit (determined or indetermined). Additionally, the weight (g) of 100 seeds of each accession was recorded. For the seed size measurements, an average of 60 seeds per accession, randomly selected, was used. Seed length (L, mm) was measured as the longest distance across the seed parallel to the hilum, seed height (H, mm) as the longest distance perpendicular to length and seed width (W, mm) was measured as the longest distance across the hilum of the seed. In addition, seed flatness (H/W), flatness index ((L+H)/2W) and elongation (L/H) were calculated. Seed shape was also characterized using the qualitative scale: rounded, oval, cuboid or kidney-shaped. Seed coat patterns were classified as absent, spotted bicolored, striped, broad striped, mottled, speckled with marginal color and colored around hilum. Seed coats colors were determined by the naked eye and only for the seeds without pattern.

In total, nine quantitative (seed length, seed width, seed height, 100-seed weight, number of seeds per pod, number of locules per pod, elongation, flatness and flatness index) and five qualitative (growth habit, seed shape, presence or absence of pattern, seed coat color and seed coat pattern) traits were measured (complete datasets available online at Figshare repository in https://doi.org/10.6084/m9.figshare.5215789.v1).

Univariate analysis of variance (ANOVA) using PROC GLM in SAS software was conducted to test the mean differences between true-type groups for the nine quantitative traits. *Post-hoc* comparisons of the accession means were carried out using Tukey's studentized range test at *P* ≤ 0.05. Pearson's correlation coefficients were calculated among the nine quantitative traits using PROC CORR and principal component analysis (PCA) was performed using PROC PRINCOMP from SAS software.

Discriminant analysis (DA) using SAS software was performed to determine which of the nine quantitative traits were the most useful for maximum discrimination among true-type groups of accessions. With DISCRIM procedure, a discriminant criterion was developed to classify each accession into one of the groups. *F*-test significance level from the analysis of covariance was used as the selection criterion. The STEPDISC procedure allowed the selection of a subset of quantitative traits for use in discriminating among the groups, through a stepwise discriminant analysis. Partial *r*^2^ statistics accounted for the significant discriminative potential of each trait, while Wilks' lambda was used to denote the statistical significance of the discriminatory power of the model function. The discriminant function, with the chosen subset of quantitative traits, was then evaluated for its performance in classifying the accessions correctly into their respective true-type group. Error rates (probabilities of misclassification) were estimated using cross-validation. The discriminant function was finally applied to the total data set (including offtype accessions), once again using the DISCRIM procedure.

For the five qualitative traits, a likelihood-ratio chi-square test in SAS software was used to test for differences in the number of accessions having a particular trait state across true-type groups. The same procedure was used to test for differences across the seven defined Portuguese regions of origin of the accessions evaluated: the north coast, northern interior, central north, central south, south, in Portugal mainland, and the two Autonomous Regions, the Azores and Madeira.

### Construction and validation of a core collection

To select a core collection that could represent the whole collection with minimum repetitiveness in future studies, we used molecular marker and morphological data. Based on the allelic diversity profile obtained in the Portuguese bean collection after the screening with 21 SSRs, one representative individual per accession was chosen. This choice relied on an individual with the most frequent genotype among each accession. Following this procedure, we used the molecular data from the 150 representative individuals of the same accessions that were phenotyped in the subsequent analysis.

The qualitative traits used were growth habit, seed shape, seed coat color, seed coat pattern, as already defined, and seed size. Seed size (qualitative) was estimated from the originally measured 100-seed weight trait, by converting it into a categorical variable, based on a classification widely used on bean by several authors for instance, Singh et al. (1991): (1) small seed size (100-seed weight < 25 g), (2) medium seed size (100-seed weight 25–40 g), and (3) large seed size (100-seed weight > 40 g).

Two different complementary algorithms were used based on a maximization (M) strategy. On the first approach, we employed the standard M strategy (Schoen and Brown, 1993) as implemented in MSTRAT (Gouesnard et al., 2001) to identify a series of nested core subsets that capture the maximum molecular and morphological diversity given the sample size. This method is considered to be the best in the coverage rate of both molecular and phenotypic data compared with the other conventional methods (Kim et al., 2007). We constructed the optimal core subset of 10 individuals (6.67% of the entire collection) and repeated the analysis for larger subsets (sample sizes: 15, 20, 25, and 30) while constraining the algorithm to include all the individuals already assigned to a core subset of a smaller size. Two hundred independent replicates and 100 iterations were generated for each sampling size and the core subset having the highest Shannon's diversity (Lewontin, 1972) was chosen. The second approach was to use the advanced maximization (M) strategy as implemented in PowerCore v1.0 (Kim et al., 2007) in order to select the accessions representing the total (100%) coverage of alleles and trait states present in the entire collection.

The molecular diversity retained in the core subsets was compared to that of the entire collection based on average number of alleles (*N*_*avg*_) as well as observed (*H*_*O*_) and expected heterozygosity (*H*_*E*_), calculated using GENEPOP v4.0. Repeated measures analysis of variance was carried out using PROC GLM in SAS. *Post-hoc* Bonferroni's adjustments were used to compare the means of diversity estimates from different core subsets, with the entire collection as control. Morphological diversity of core subsets and of the entire collection was assessed by calculating the average number of traits states and Shannon's diversity index (*H*_*Sh*_). The principal coordinate analysis (PCoA) based on molecular data was performed using NTSYS-pc v2.10 (Rohlf, 2005) to graphically represent genetic relationships among bean accessions. The proportion-of-shared-alleles distances (*D*_*PSA*_) (Bowcock et al., 1994) among representative individuals of each accession were calculated using MICROSAT (Minch et al., 1997).

## Results

### Allelic diversity

The microsatellite markers screening of the 175 Portuguese accessions, and 17 Andean and Mesoamerican race representatives, and wild bean relatives identified 225 alleles (complete dataset available online at Figshare repository in https://doi.org/10.6084/m9.figshare.5215789.v1). All 21 SSRs analyzed were polymorphic with an average of 10.71 alleles per locus, varying from 2 to 26 alleles each (Supplementary Table 3). Overall, the most informative markers (with the highest PIC) were PVat007, GATS91, and BM143, which were also the ones showing the highest number of alleles per locus. Within the Portuguese accessions, 188 alleles were identified, with an average of 8.95 alleles per locus, and 39 private alleles were found. Within race representatives and wild relatives, 151 alleles were identified, with an average of 7.19 alleles per locus, and 26 private alleles were found (Supplementary Table 3). The effective number of alleles (*Ne*) was 1.113 for both groups (Table 1). Also, there were no significant differences in allelic richness (*N*_*ar*_) among the two groups: 1.098 in the Portuguese accessions and 1.100 in the race representative and wild relative bean accessions [*P*_(*KW*)_ = 1.000]. Private allelic richness (*N*_*par*_) was on average 0.005 for the Portuguese accessions and 0.078 for Andean and Mesoamerican race representatives and wild bean relatives. Observed heterozygosity (H_*O*_) was low for both groups, as expected in a mainly self-pollinated species, but significantly lower [*P*_(*KW*)_ = 0.018] for the Portuguese accessions, with an average value of 0.027 in the Portuguese accessions and of 0.067 in the race representatives and wild relatives (Table 1). Sixty-two Portuguese accessions had no heterozygous individuals (H_*O*_ = 0.000) and nine of them were genetically uniform (H_*E*_ = 0.000). The average value of H_*E*_ was equal for the Portuguese bean accessions and for the race representatives and wild relatives, with no significant differences among groups [*P*_(*KW*)_ = 0.920] at the accession level (Table 1).

**Table 1:** Genetic diversity of the common bean accessions as assessed by 21 microsatellite loci.

**Parameter**	**Origin of accessions**				**P(KW)^a^**
	**Portugal**		**Andean and mesoamerican race representatives and wild bean relatives**		
	**Average^b^**	**Range**	**Average**	**Range**	
No. accessions	175		17		
Number of alleles (*N_*a*_*)	1.337	(1.00–2.52)	1.331	(1.00–1.81)	
Allelic richness (*N_*ar*_*)	1.098	(1.00–1.50)	1.100	(1.00–1.25)	1.000
Effective number of alleles (*N_*e*_*)^c^	1.113	(1.00–2.12)	1.113	(1.00–1.35)	
Number of private alleles (*N_*pr*_*)	0.223	(0.00–3.00)	1.529	(0.00–8.00)	
Total number of private alleles	39		26		
Private allelic richness (*N_*par*_*)	0.005	(0.00–0.09)	0.078	(0.00–0.33)	
Observed heterozygosity (*H_*O*_*)	0.027	(0.00–0.23)	0.067	(0.00–0.20)	0.018
Expected heterozygosity (*H_*E*_*)	0.102	(0.00–0.53)	0.102	(0.00–0.26)	0.920

a *Significance of Kruskal-Wallis test*.

b *Average (and range) across accessions*.

c *Harmonic mean*.

The diversity parameters *N*_*ar*_, *H*_*O*_, and *H*_*E*_ between the two groups (Portuguese vs. Andean and Mesoamerican race representatives and wild bean relatives), tested on individual level across microsatellite markers, showed that *H*_*O*_ was significantly different [*P*_(*KW*)_ = 0.026] among groups. No significant differences [*P*_(*KW*)_ = 0.479] were found on *N*_*a*_. In contrast, *H*_*E*_ at the individual level was significantly higher [*P*_(*KW*)_ = 0.000] for the Andean and Mesoamerican race representatives and wild bean relatives than for the Portuguese accessions (Supplementary Table 4).

### Genetic diversity across regions

The genetic diversity analysis of the Portuguese common bean accessions grouped by region of origin, as assessed by 21 SSRs, is depicted in Supplementary Table 5. Kruskal-Wallis test indicated that no significant differences were found on genetic diversity parameters such as allelic richness [*P*_(*KW*)_ = 0.506], observed [*P*_(*KW*)_ = 0.147] and expected heterozygosity [*P*_(*KW*)_ = 0.476] among accessions from different regions of origin.

### Isolation by distance analysis

The isolation by distance analysis among the 134 Portuguese common bean accessions tested revealed that there was no relationship between the geographic distance and the genetic differentiation of the accessions (*r* = 0.0054; *P*_Mantel_ = 0.44, Supplementary Figure 5).

### Phaseolin types

Three phaseolin patterns (classified here as haplotypes P1, P2, and P3) were the most common among accessions after screening with the molecular marker for the phaseolin-type analysis. Comparing the haplotypes found in the Portuguese accessions with the ones from the bean gene pool representatives, it was possible to relate the patterns obtained with the phaseolin types already described by Gepts et al. (1986) for the Mesoamerican and Andean gene pools. The accessions representative of Mesoamerican gene pool analyzed were from ecogeographic races *Jalisco, Guatemala*, and *Mesoamerica* and had phaseolin types S and/or B; while accessions representative of Andean gene pool analyzed were from the ecogeographic races *Peru, Chile*, and *Nueva Granada* and had phaseolin types H, C, or T, according to CIAT gene bank information.

Thus, the pattern obtained for P1 haplotype corresponded to S type phaseolin characteristic of Mesoamerican accessions, while the pattern obtained for P2 haplotype corresponded to H or C types of phaseolin and the pattern obtained for P3 haplotype corresponded to T type of phaseolin, both characteristic of the Andean accessions (Supplementary Table 6).

### Analysis of molecular variance

AMOVA was conducted to test for the existence of genetic structure between and within groups of accessions (Portuguese accessions vs. gene pool representatives and wild relatives from the CIAT collection). This analysis showed that the differences between the two groups of accessions (Portuguese and CIAT accessions) were significant (*P* < 0.0001) and explained 12.9% of the total genetic variance (Table 2). For the entire set of analyzed accessions, the major source of variance was among accessions and not within them (83.6 vs. 16.4%), reflecting again the predominant self-pollinating reproductive system of bean. Similar percentages were observed when the Portuguese accessions were analyzed separately from the race representative and wild relative accessions (Table 2).

**Table 2:** Analysis of molecular variance for the partitioning of microsatellite diversity.

**Analysis**	**Source of variation**	**df**	**Variance components**	**% Total variance**	**ϕ-statistics**	***P (ϕ)***
(A) All accessions	Among accessions	191	4.311	83.65	0.837	<0.0001
	Within accessions	3,460	0.842	16.35		
(B) Portuguese vs. race representative and wild relatives	Among groups	1	0.746	12.89	0.129	<0.0001
	Among accessions within groups	190	4.197	72.55	0.833	<0.0001
	Within accessions	3,460	0.842	14.56	0.854	<0.0001
(C) Portuguese	Among accessions	174	4.093	83.11	0.831	<0.0001
	Within accessions	3,175	0.832	16.89		
(D) Race representative and wild relatives	Among accessions	16	5.416	84.89	0.849	<0.0001
	Within accessions	285	0.964	15.11		

*(A) among and within all bean accessions, (B) among groups of accessions (Portuguese vs. race representative and wild relatives), among accessions within groups and within accessions, as well as among and within accessions, separately for Portuguese (C) accessions and for race representative and wild relative accessions (D)*.

### Genetic analysis and population structure

**Figure 1:**
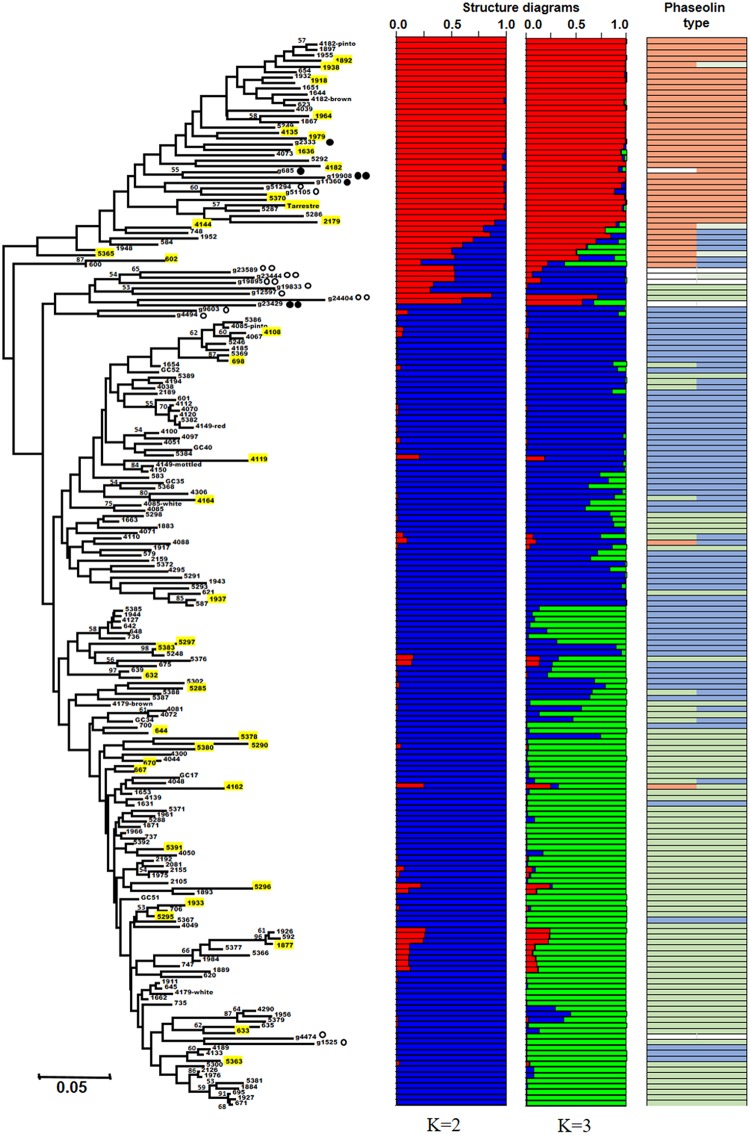
Neighbor-joining tree based on chord distance between 192 bean accessions (175 Portuguese and 17 Mesoamerican and Andean race representatives and wild bean relatives). The 37 accessions belonging to the core collection are highlighted in yellow. *Structure* bar plots of average proportions of membership for K = 2 (in red and blue) and K = 3 (in red, blue, and green) clusters are given for all the accessions studied. Bootstrap values above 50 are shown. The most frequent phaseolin haplotypes identified in the accessions are indicated in the last column with a color code (P1 in orange, P2 in light green, and P3 in light blue); Other types of phaseolin patterns are colored white. 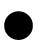 accession representative of the Mesoamerican domesticated gene pool; 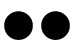 wild accession from the Mesoamerican region; 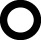 accession representative of the Andean domesticated gene pool; 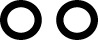 wild accession from the Andean region.

The average Cavalli-Sforza and Edwards' chord distance, generated from the SSR data, was 0.193 among Portuguese accessions, while it was 0.258 for the race representatives and wild relatives (Table 3 and Figure 1). Although the maximum genetic distance was similar for the two groups of accessions (0.394 for the Portuguese accessions, between accessions 1892 and 5302 as well as between accessions 1938 and 5302, vs. 0.389 for the Andean and Mesoamerican race representatives and wild relatives, between accessions g2333 and g9603), the minimum values were substantially lower among Portuguese accessions (0.003, between accessions 1976 and 2126, vs. 0.102 for the race representatives and wild accessions, between accessions g51105 and g51294). Three major clusters were visualized in the Neighbor-joining tree (Figure 1). One cluster grouped mainly the race representatives and wild relatives from the Mesoamerican region, with a small part of the Portuguese accessions. The other two clusters grouped most of the Portuguese accessions with the race representatives and the wild relatives from the Andean regions.

**Table 3:** Pairwise Cavalli-Sforza and Edwards' chord distances among the analyzed bean accessions.

**Origin of accessions**	***D_*Chord*_***		
	**Average**	**Min**	**Max**
Portugal	0.193	0.003	0.394
Andean and Mesoamerican race representatives and wild accessions	0.258	0.102	0.389
All accessions	0.205	0.003	0.398

Structure analysis of the bean accessions analyzed confirmed the major clusters found using the Neighbor-joining algorithm. The highest ΔK was observed for K = 2 (7028.15) followed by K = 3 (657.21). For K higher than 3, ΔK was always inferior to 2.9. K = 2 showed the major separation between Andean and Mesoamerican gene pools, while K = 3 showed an additional sub-division within the Andean gene pool, separating the representatives of the races *Peru* and *Nueva Granada* from the ones of *Chile*. The Portuguese accessions distributed along with the analyzed representative accessions from both Mesoamerican and Andean gene pools but with a higher prevalence closer to the Andean clusters. Through the combination of the structure analysis with the three major phaseolin haplotypes found (P1, P2, P3), we observed that cluster A (red) contains most of the accessions with P1 phaseolin type, characteristic of the Mesoamerican gene pool, and clusters B1 (blue) and B2 (green) most of the accessions with P3 and P2 phaseolin types, respectively, both characteristic of the Andean gene pool (Figure 1).

### Genetic differentiation into subgroups

Based on the classification made, using the results of structure analysis and phaseolin haplotype/cluster membership matching, different groups of accessions were established. From the total of 175 Portuguese accessions, 118 were classified as true-type accessions. Those were distributed in the three true-type groups as follows: 26 accessions in the AP1 group, 36 accessions in the B1P3 group and 56 accession in the B2P2 group. The remaining 57 accessions (33%) were not assigned to any gene pool since they represented putative hybrids among gene pools hereby referred as offtypes (Table 4). Within the offtype accessions, 7 were classified as composite, 20 as hybrid and 30 as non-corresponding.

**Table 4:** Assignment of the Portuguese bean accessions into subtypes according to the SSR analysis and phaseolin haplotype.

**Classification**	**Membership cluster**	**Phaseolin haplotype**	**No accessions**	**% accessions**
True-type	A	P1 - S Mesoamerican type	26	14.86
True-type	B1	P3 - T Andean type	36	20.57
True-type	B2	P2 - H, C Andean type	56	32.00
Offtypes	–	–	57	32.57
Total			175	100

Among the true-type groups of accessions (AP1, B1P3, B2P2), a Kruskal-Wallis test showed that the differences between the average values of N_*ar*_,H_*E*_, and H_*O*_ were statistically significant (*p* < 0.05). A Wilcoxon test (between all possible pairs of groups) revealed that the significant differences found were between the AP1 and both B1P3 and B2P2 groups; that is, between the Mesoamerica and Andean groups (Table 5).

**Table 5:** Number of bean accessions (n), mean values of allele richness (N_*ar*_), observed (H_*o*_) and expected heterozygosity (H_*E*_) distributed in the different Portuguese bean group types.

**Accession group type**	**Subtype**	***n***	**N_*ar*_**	**Wilcoxon test^a^**	**H_*O*_**	**Wilcoxon test**	**H_*E*_**	**Wilcoxon test**
True-type	AP1	26	1.116	a	0.088	a	0.116	a
	B1P3	36	1.065	b	0.007	b	0.068	b
	B2P2	56	1.078	b	0.012	b	0.080	b
P (Kruskal-Wallis)			0.002		0.000		0.005	
Offtype	Composite	7	1.356		0.040		0.374	
	Hybrid	20	1.083		0.017		0.085	
	Non-corresponding	30	1.111		0.029		0.116	
	All	175	1.098		0.027		0.102	

*Kruskal-Wallis test is shown for true-types only. Letters a and b reflect the result from the Wilcoxon test*.

a *Different letters in the same column indicate significant differences between values at P < 0.05 on the basis of the Wilcoxon test*.

### Seed and plant morphological traits

High diversity was found among the 150 bean accessions analyzed for the nine quantitative traits (seed length, seed width, seed height, seed elongation, seed flatness, seed flatness index, 100-seed weight, number of seeds per pod and number of locules per pod) measured. The analysis of variance revealed significant differences among bean true-type groups AP1, B1P3, and B2P2 for the nine quantitative traits evaluated (Supplementary Table 7).

Tukey's studentized test revealed that the three true-type bean groups were significantly different from each other in seed width and flatness. Accessions from the AP1 group were significantly lighter (inferior 100-seed weight) than the accessions from the other groups. Accessions from the B2P2 group had a significantly larger seed height and a lower flatness index, than the rest. Accessions from the B1P3 group had significantly larger seed length, with higher seed elongation, but with a smaller number of seeds per pod as well as a smaller number of locules per pod, than the rest of the accessions from the other true-type groups.

Besides the nine quantitative traits, five qualitative traits were evaluated on the Portuguese bean accessions. True-type groups were tested for significant differences among trait states using the likelihood-ration chi-square test.

Seed shapes were categorized into four different classes: round, oval, cuboid and kidney shape (Table 6).

**Table 6:** Seed shapes found in the collection of 150 bean seeds distributed by accession group types.

**Accession group type**	**Subtype**	**Seed shape**				
		**Round**	**Oval**	**Cuboid**	**Kidney**	**Total**
True-type	AP1	1	1	15	4	21
	B1P3	0	1	17	7	25
	B2P2	0	22	22	6	50
	*P*(χ^2^)			0.0001386		
Offtype	Composite	0	3	1	2	6
	Hybrid	0	4	6	9	19
	Non-corresponding	0	10	14	5	29
	Total	1	41	75	33	150

*Significance of the likelihood-ratio chi-square, among true-type groups, is shown*.

Half of the 150 Portuguese accessions analyzed possesses a cuboid seed shape, followed by the oval (27.3%) and the kidney-shaped (22%). The cuboid shape was the most frequent seed shape in all three true-type groups. Nonetheless, significant differences were found among the three true-type groups within the number of accessions having a particular seed shape trait state. For instance, accessions from the true-type B2P2 group clearly showed a much higher percentage of oval seeds and a lower percentage of cuboid or kidney-shaped seeds, when compared with the other two true-type group accessions.

From the total of 150 Portuguese bean accessions evaluated, 90 presented plain coat seeds (no seed coat pattern) and 60 presented a patterned seed coat. The chi-square test indicated that there were no significant differences in the number of accessions having or not a seed pattern among the true-type groups [*P*_(χ2)_ = 0.563] (Supplementary Table 8).

Within the 60 accessions analyzed that had a seed coat pattern, six different patterns, also described in IBPGR (1982), were found (Table 7).

**Table 7:** Pattern types of the 60 Portuguese bean accessions analyzed exhibiting seed coat patterns, distributed according to accession group types.

**Accession group type**	**Subtype**	**Seed coat pattern**					
		**Spotted bicolor**	**Speckled with marginal color**	**Striped**	**Mottled**	**Broad striped**	**Colored around hilum**
True-type	AP1	0	1	6	0	0	0
	B1P3	0	0	8	0	3	1
	B2P2	2	2	17	2	0	0
	*P*(χ^2^)			0.0581			
Offtype	Composite	0	0	1	0	0	0
	Hybrid	0	1	4	1	1	0
	Non-corresponding	4	0	5	1	0	0
	Total	6	4	41	4	4	1

*Significance of the likelihood-ratio chi-square is shown among true-type groups*.

The most common seed coat pattern observed among Portuguese accessions was striped. In accordance with the chi-square test performed, no particular true-type group stood out with a significant different number of accessions having a particular seed coat pattern.

Eight different seed coat colors were attributed by the naked eye to the plain coat seeds (with no pattern): white, yellow, pink, light brown, brown, red, purplish red and black (Table 8).

**Table 8:** Seed coat colors of the 90 Portuguese bean accessions analyzed with plain seed coat (no patterned seed coat), distributed by accession group types.

**Accession group type**	**Subtype**	**Seed coat color (plain seed coat)**							
		**white**	**yellow**	**pink**	**light brown**	**brown**	**red**	**purplish red**	**black**
True-type	AP1	7	0	1	2	3	0	0	1
	B1P3	4	1	2	0	0	5	1	0
	B2P2	1	0	10	3	6	6	1	0
	*P*(χ^2^)				0.0007				
Offtype	Composite	3	1	0	0	0	1	0	0
	Hybrid	5	2	2	1	1	1	0	0
	Non-corresponding	12	0	1	1	4	0	1	0
	Total	32	4	16	7	14	13	3	1

*Significance of the likelihood-ratio chi-square is shown among true-type groups*.

The four most frequent colors of plain seed coats among the Portuguese beans were white, pink, brown and red. For this trait, there were significant differences among the true-type groups of accessions [*P*_(χ2)_ = 0.0007]. Accordingly, the AP1 group of accessions had no red-colored accessions and most of its accessions were white, whereas both the B1P3 and B2P2 groups presented red seed coat accessions. Moreover, the majority of the accessions from the B2P2 group were pink.

Regarding the plant growth habit of the 150 Portuguese bean accessions, 92 accessions had a determinate growth habit, and 58 an indeterminate growth habit (Table 9).

**Table 9:** Plant growth habit of 150 Portuguese bean accessions.

**Accession group type**	**Subtype**	**Plant growth habit**	
		**Determinate**	**Indeterminate**
True-type	AP1	12	9
	B1P3	25	0
	B2P2	20	30
	*P*(χ^2^)	9.15 × 10^−8^	
Offtype	Composite	4	2
	Hybrid	14	5
	Non-corresponding	17	12
	Total	92	58

*Significance of the likelihood-ratio chi-square is shown among true-type groups*.

Significant differences among true-type groups were observed. All the accessions from the B1P3 group had a determinate growth habit, whereas the other two true-type groups had accessions with both growth habits. Nevertheless, the AP1 group had a prevalence of determinated plants, while among the B2P2 group the more common growth habit was the indetermined one. Among offtype groups most of the accessions had determinated plants.

There were no significant differences among the true-type groups in the number of accessions collected in each of the seven different Portuguese regions of origin (north coast, northern-interior, central north, central south, south, the Azores, Madeira) [*P*_(χ2)_ = 0.316] (Supplementary Table 9). The larger proportion of accessions came from the north central and north interior regions (39 and 35%, respectively).

Moreover, there were not significant differences in the distribution of accessions from each region of origin when comparing true-type and offtype groups [*P*_(χ2)_ = 0.211].

### Correlation between the quantitative morphological traits

The estimation of the Pearson's correlation coefficients among the nine quantitative morphological traits allowed the measurement of dependence between traits. Accordingly, seed length was highly correlated with 100-seed weight (*r* = 0.706), elongation (*r* = 0.841) and flatness index (*r* = 0.759). Not surprisingly, there were also strong positive correlations between the number of locules per pod and the number of seeds per pod (*r* = 0.773), between elongation and flatness index (*r* = 0.771) and between flatness and flatness index (*r* = 0.740). On the other hand, there was a strong negative correlation between flatness index and seed width (*r* = −0.704) (Supplementary Table 10).

### Principal components analysis

A principal components analysis was performed to assess the relationship among the accessions and to visualize if the different group types, previously defined, cluster separately, based on the results from nine quantitative traits (seed length, seed width, seed height, seed elongation, seed flatness, seed flatness index, 100-seed weight, number of seeds per pod and number of locules per pod).

**Figure 2:**
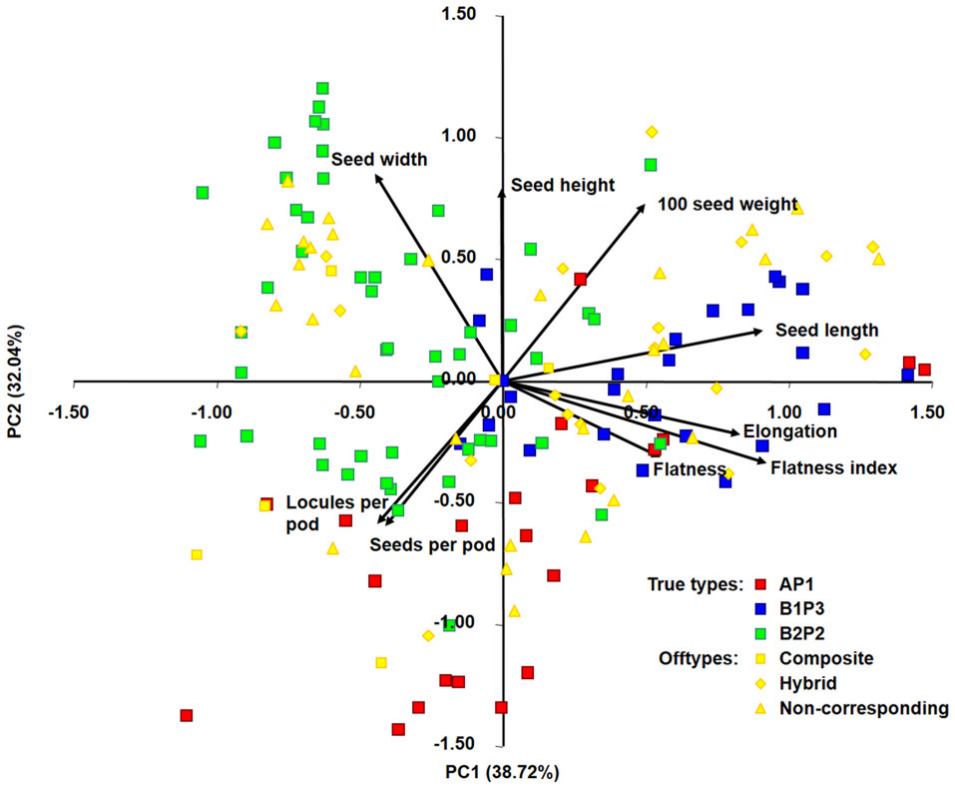
Principal components analysis of 150 Portuguese bean accessions based on nine quantitative morphological traits. Ninety-six accession assigned as true-types (AP1 in red, B1P3 in blue, B2P2 in green) and 54 accessions assigned as offtypes (composite, hybrid, non-corresponding) in yellow. A, B1, and B2 are the same clusters from structure analysis; P1, P2, and P3 are the phaseolin haplotypes identified.

The biplot projecting the 150 bean accessions (96 true-type and 54 offtype) and the nine quantitative morphological traits was constructed using the two first principal components explaining 70.8% of the variance found among accessions (Figure 2). PC1 differentiates primarily B1P3 (in blue) from B2P2 (in green), with the main contributions of traits like seed length, elongation and flatness index. PC2 differentiates AP1 (in red) from both B1P3 and B2P2, with the main contributions of traits like seed width, seed height and 100-seed weight. The offtype accessions (in yellow) were positioned among the true-types without any clear distribution pattern.

### Discriminant analysis

The stepwise discriminant analysis revealed that seed width, seed elongation, number of locules per pod, seed flatness and seed height were, in this order and in accordance to partial *r*^2^ statistics, the most useful traits in discriminating accessions among the three true-types (Table 10).

**Table 10:** Stepwise discriminant analysis summary for the five most informative traits allowing maximum discrimination among bean accessions from true-type groups (AP1, B1P3, B2P2).

**Trait**	**Partial *r*^2^**	***F*-value**	**P(F)**	**Wilks' λ**	**P(λ)**
Seed width (mm)	0.521	50.54	^***^	0.479	^***^
Elongation (L/H)	0.387	29.05	^***^	0.294	^***^
Locules per pod	0.265	16.43	^***^	0.216	^***^
Flatness (H/W)	0.119	6.08	^**^	0.190	^***^
Seed height (mm)	0.120	6.05	^**^	0.167	^***^

P-value significant level:

** *0.001 < p value < 0.01*,

*** *p value < 0.001*.

These five traits were tested for their performance, and 86.5% of the accessions were correctly classified into their respective true-type groups. Thirteen accessions (out of the 96 with a true-type associated) were misclassified (data not shown).

**Figure 3:**
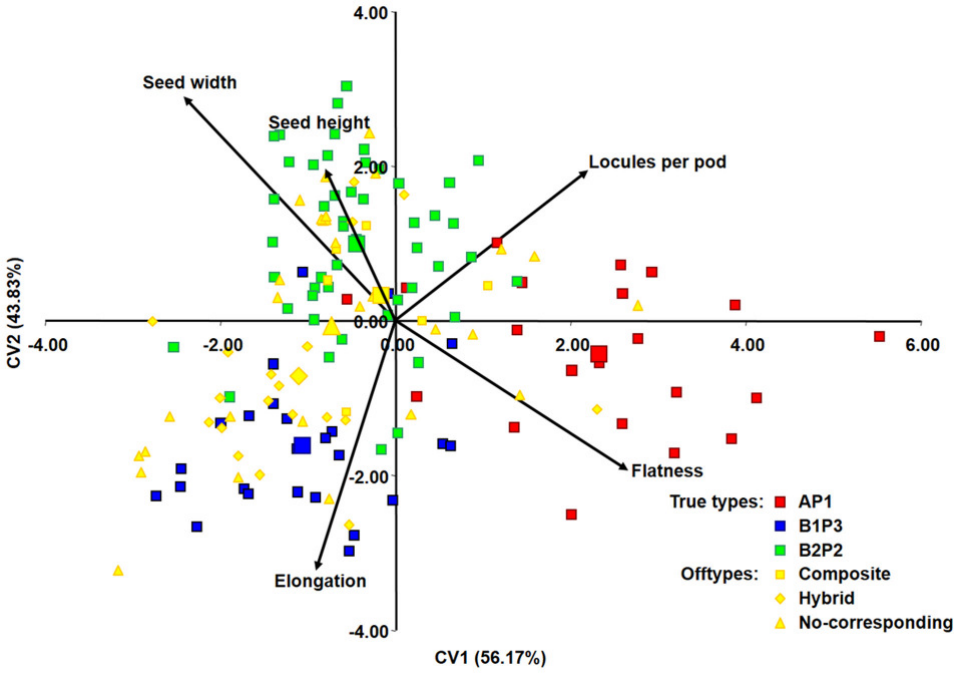
Discriminant analysis applied to 150 Portuguese bean accessions based on the five quantitative morphological traits that were the most useful for maximum discrimination between true-type groups (AP1, B1P3, B2P2). A, B1, and B2 are the same clusters from structure analysis; P1, P2, and P3 are the phaseolin haplotypes identified. Larger data point marks signal the center of the distribution of different groups.

The discriminant function, resulting from these five traits, was applied to the total data set (including offtype accessions) resulting in a clear separation among the true-type groups, with offtype accessions in intermediate positions (Figure 3).

### Core collection development

To form core collections based on 150 individual genotypes representing the Portuguese bean germplasm, molecular (21 microsatellites, 187 different alleles) and morphological data (five qualitative traits, with 25 different states) were used. The five qualitative traits used were growth habit, seed shape, seed coat color, seed coat pattern and seed size.

Of the Portuguese accessions analyzed, 73.3% were considered of large seed size (100-seed weight >40 g), 23.3% of medium size (100-seed weight 25–40 g) and 3.3% of small size (100-seed weight < 25 g) (Supplementary Table 11).

With the standard M strategy as implemented in MSTRAT (Gouesnard et al., 2001), five nested core subsets (sample size: 10, 15, 20, 25, 30) were first built. Next, the advanced M strategy as implemented in PowerCore v1.0 (Kim et al., 2007) allowed the selection of a core set of 37 accessions, representing the smallest set of accessions with the total coverage of alleles and trait states present in the entire collection.

The composition of each core set is shown in Table 11.

**Table 11:** Number of bean accessions from core subsets belonging to each group type.

**Accession group type**	**Subtype**	**Entire collection**	**Core 37**	**Core 30**	**Core 25**	**Core 20**	**Core 15**	**Core 10**
True-type	AP1	21	10	11	10	7	6	3
	B1P3	25	6	6	5	4	2	2
	B2P2	50	10	5	4	4	3	3
Offtype	Composite	6	2	1	1	1	0	0
	Hybrid	19	4	2	2	2	2	1
	Non-corresponding	29	5	5	3	2	2	1
	Total	150	37	30	25	20	15	10

**Figure 4:**
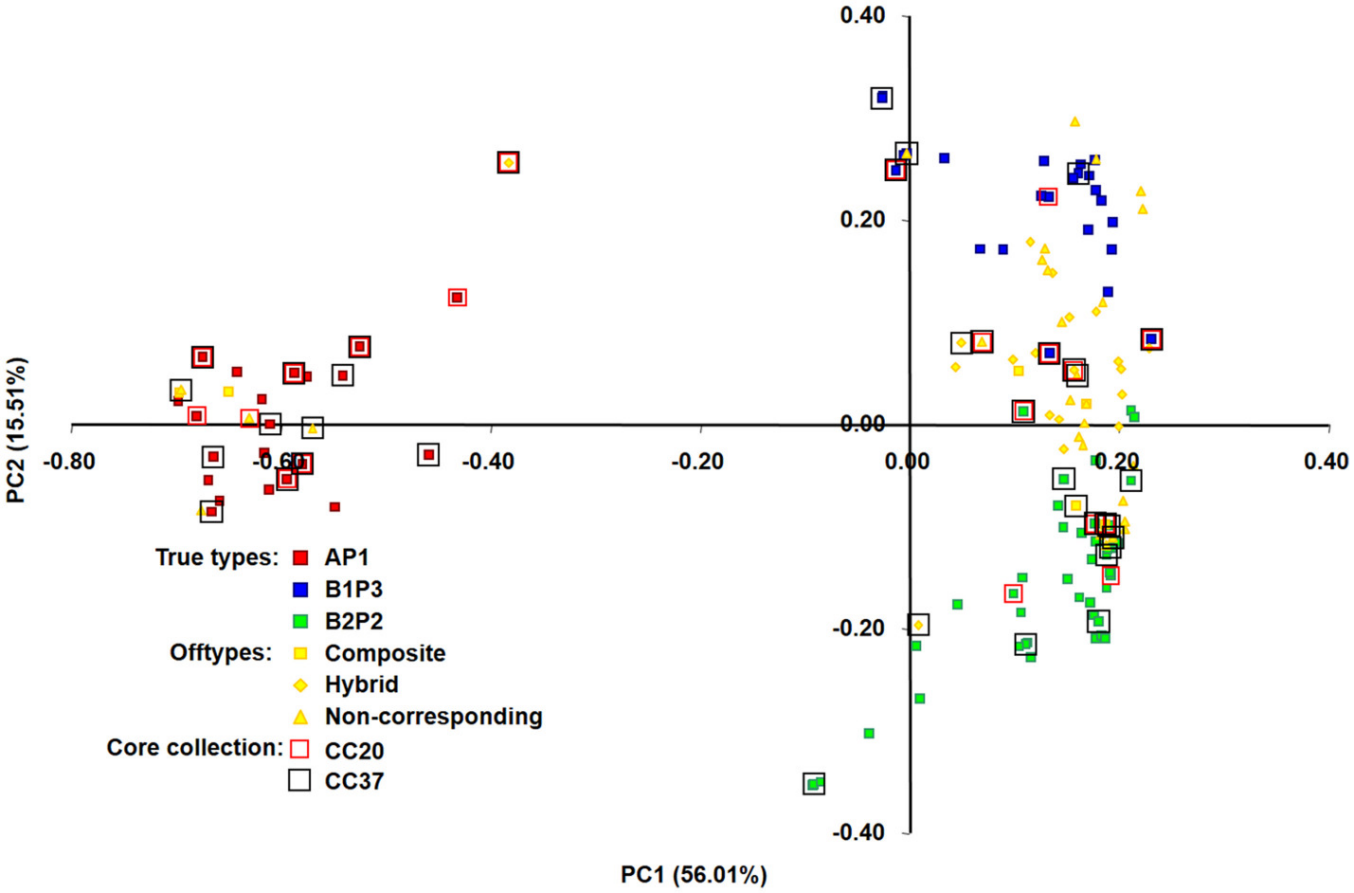
Principal co-ordinate analysis (PCoA) of 150 bean accessions based on proportion-of-shared-alleles distance matrix. 71.52% of the genetic variation in the Portuguese accessions was explained by the first two axes. A, B1, and B2 are the same clusters from structure analysis; P1, P2, and P3 are the phaseolin haplotypes identified. Accessions included in core collections with 20 and 37 accessions are highlighted by red (CC20) and black (CC37) squares, respectively.

When comparing the composition of each core set with the entire collection, an overrepresentation of accessions from the AP1 group (Mesoamerican) was observed. Accessions from this group represented 14% of the entire collection while, for instance, in core sets with 37 and 20 accessions, this percentage increased to 27 and 35%, respectively (Figure 4).

Molecular and morphological diversity of the entire collection was compared with the core subsets (Table 12). The core collection with 20 accessions is the smallest set, with a non-significantly lower average number of alleles in comparison with the entire collection. Regarding the morphological characterization based on the five qualitative traits, even the core set with only 10 accessions, capturing 80% of these traits states diversity, had an average number of trait states and a Shannon's diversity index not significantly different from the entire collection. The core set with 37 accessions contained 100% of the allelic and morphologic diversity. This core collection was constituted by 10 accessions belonging to the AP1 group (Mesoamerican), 6 accessions from the B1P3 group (Andean) and 10 accessions from the B2P2 group (Andean). The remaining 11 accessions were offtypes: 2 composite accessions, 4 hybrid accessions and 5 non-corresponding accessions (Figure 4).

**Table 12:** Comparison between the entire collection and core subsets of molecular and morphological diversity parameters, based on 21 SSRs and 5 qualitative traits.

**Data**	**Set**	**Entire collection**	**Core37**		**Core30**		**Core25**		**Core20**		**Core15**		**Core10**	
	N_*ind*_	150	37		30		25		20		15		10	
	N_*ind*_(%)	100.00	24.67		20.00		16.67		13.33		10.00		6.67	
Molecular diversity	N_*a*_	134	134		129		125		121		112		98	
	N_*a*_(%)	100.00	100.00		96.27		93.28		90.30		83.58		73.13	
	N_*avg*_	6.381	6.381	ns	6.143	ns	5.952	ns	5.762	ns	5.333	^**^	4.667	^***^
	H_*O*_	0.020	0.039	ns	0.051	^**^	0.052	^**^	0.048	^**^	0.055	^**^	0.043	ns
	H_*E*_	0.512	0.624	^***^	0.653	^***^	0.663	^***^	0.662	^***^	0.682	^***^	0.671	^***^
Morphological diversity	N_*s*_	25	25		24		23		22		21		20	
	N_*s*_(%)	100.00	100.00		96.00		92.00		88.00		84.00		80.00	
	Ns_*avg*_	5.000	5.000	ns	4.800	ns	4.600	ns	4.400	ns	4.200	ns	4.000	ns
	H_*Sh*_	1.514	1.621	ns	1.577	ns	1.548	ns	1.549	ns	1.560	ns	1.606	ns

P-value significant level:

** *0.001 < p value < 0.01*,

*** *p value < 0.001*.

*ns, not significant (p-value > 0.05)*.

*N_ind_, no. of individuals; N_a_, no. of alleles; N_avg_, average no. of alleles per locus; H_o_, observed heterozygosity; H_E_, expected heterozygosity; N_s_, no. of trait states captured in the core set; Ns_avg_, average no of trait states; H_Sh_, Shannon's diversity index*.

To graphically represent the genetic relationships among the Portuguese bean accessions belonging to the different core sets built, a principal co-ordinate analysis (PCoA), based on proportion-of-shared-alleles distance matrix, was performed. Two axes were generated with a relatively large eigenvalue, capturing 72.03% of the variation in our data (Figure 4). A clear separation of the three true-type groups (AP1 in red, B1P3 in blue and B2P2 in green) was observed. PC1 separated primarily AP1 (Mesoamerican group) from the two Andean types, while PC2 separated the two Andean groups (B1P3 and B2P2). Core sets were scattered along the plot with accessions from all the different group types, but with an overrepresentation of the Mesoamerican group, as was previously noticed.

## Discussion

To enrich the current knowledge of molecular and morphological diversity of the Portuguese bean germplasm, we performed, for the first time, a simultaneous molecular marker screen and seed and plant morphological characterization of representative accessions of all national bean-growing traditional regions. Moreover, this bean collection was compared at the molecular level with representatives of the Andean and Mesoamerican gene pools. On the basis of these data, core subsets of the Portuguese germplasm were developed. High genetic and morphological diversity was detected among accessions of this collection. The majority of the Portuguese accessions analyzed were more related to the Andean gene pool. From the entire collection, 37 accessions were selected to form a core collection that retains all the diversity within this germplasm, with minimum repetitiveness. This subset of accessions will allow an increased efficiency of the forthcoming more detailed characterization of this valuable resource. This information is vital to support a more effective conservation of the Portuguese bean germplasm and to promote its use in both national and international breeding programs of this important crop.

The study of the genetic structure and phaseolin type diversity of the collection showed its extent of admixture and the bean gene pool more related to the Portuguese germplasm. One- third of the accessions evaluated were of an intermediate nature. By examining the position of the Portuguese accessions on the Neighbor-joining tree, we conclude that most of the national germplasm is genetically closer to the Andean gene pool, despite the existence of accessions that were distributed with representatives from both gene pools (Mesoamerican and Andean). The analysis of genetic structure and phaseolin types divided the collection into three main clusters: 14.8% of accessions into type AP1 (genetically more related to the Mesoamerican gene pool representatives), 20.6% of accessions into type B1P3 and 32% of accessions into type B2P2 (both genetically more related to the Andean gene pool representatives). The remaining 32.6% of the accessions had an intermediate or admixture nature and were classified as offtypes.

The predominance of Andean bean germplasm found in this Portuguese collection was also reported for other *P. vulgaris* collections from all over Europe, from the Mediterranean countries Portugal, Spain, France and Italy (Rodiño et al., 2003; Sicard et al., 2005; Raggi et al., 2013), to central northern, eastern and southeastern countries (Gepts and Bliss, 1988; Logozzo et al., 2007; Angioi et al., 2010, 2011). This Andean relationship was further supported by the analysis of phaseolin types, with 77% of the Portuguese accessions having a phaseolin pattern attributable to the Andean gene pool, and by the seed size analysis, in which 73% of the Portuguese accessions depicted large seeds, also characteristic of Andean races (Singh et al., 1991; Blair et al., 2007). When data was graphically represented using principal component analysis (Figure 3), it was clear that higher values of seed width and seed height were found in seeds from the B2P2 group; more elongated seeds belonged to the B1P3 groups, and seeds flatter and with a higher number of locules per pod were predominant in the AP1 group. This evidence is in accordance with previous studies that characterized seeds from the Andean gene pool as larger-sized and accessions from Mesoamerica as higher-yielding but with smaller seeds (Singh et al., 1991, 1993; Kelly et al., 1998; De Ron, 2015). Moreover, the plant growth habit of more than 61% of the Portuguese accessions was determinate, which is mostly typical of the *Nueva Granada* Andean race (Singh et al., 1991). Interestingly, the genetic structure and phaseolin pattern analysis performed was also in accordance with the separation of major known races within the representatives from the Andean gene pool. Thus, cluster B1P3 contained the representatives from races *Nueva Granada* and *Peru*, whereas cluster B2P2 contained accessions from race *Chile*.

The considerable percentage of offtype accessions (32.6%), with novel genetic combinations not typical from the primary centers of domestication, emphasize the potential value of the Portuguese germplasm, providing an additional level of complexity and new gene combinations not yet explored in breeding. In fact, introgression between the Mesoamerican and Andean gene pools had already been detected in the Iberian Peninsula using phaseolin, allozymes and morphological data (Santalla et al., 2002; Rodiño et al., 2006). During this gene flow, new and interesting combinations of traits, such as higher adaptability to environmental stresses, disease resistance or seed quality, may have arisen. In addition, the negative correlation between seed weight and yield potential may have been overtaken (White and González, 1990), with a possible increment of yield among the large-sized Andean germplasm. The existence of a greater proportion of admixed accessions in Europe than in America was already reported by González et al. (2009), who estimated that the percentage of intermediate genotypes in Europe is around 40%, whereas in the Americas it is 12%. Intermediate forms were also previously observed in accessions from the Iberian Peninsula that was considered a secondary center of genetic diversity of bean (Santalla et al., 2002). However, in that study, only accessions from the north of Portugal were analyzed.

The analysis of molecular diversity using 21 microsatellite markers showed that the Portuguese bean germplasm contains a high level of genetic diversity, with 188 alleles found, an average of 9 alleles per locus, and 39 private alleles. Those values were initially considered higher than the ones obtained for the representatives of Mesoamerican and Andean gene pools and wild relatives: 151, 7, and 26, respectively. Nevertheless, when sample size was taken into account, allele richness was identical in both groups (1.1). Similar genetic diversity values for these microsatellites were observed by other authors, in studies with wild and domesticated bean accessions from the Andean and Mesoamerican gene pools (Kwak and Gepts, 2009; Blair et al., 2012), and with accessions from Brazil, also considered a secondary center of diversity (Burle et al., 2010). In our work, the average value of observed heterozygosity was 0.027 for the Portuguese accessions and 0.067 for the Andean and Mesoamerican gene pool representatives and wild relatives. These low values are characteristic of predominantly self-pollinating crops such as bean (Papa et al., 2007). In accordance, the AMOVA results revealed that 83.65% of the overall variance found was among accessions and only 16.35% within accessions.

The presence of many intermediate forms in the collection was also observed at seed level, hampering the seed classification according to the traditional market classes (Voysest and Dessert, 1991). The high morphological diversity of Portuguese bean seeds had already been reported in previous studies with a smaller number of accessions (Gil and Ron, 1992; Rodiño et al., 2001; Santalla et al., 2002; Freitas et al., 2010). Among the accessions studied here, eight different seed coat colors, six seed coat patterns and four seed shapes were detected, once more highlighting the variability within this germplasm. Still, the colors (white, pink, brown and red), shapes (cuboid, oval and kidney) and patterns (absent and striped) more frequent in the Portuguese germplasm were among the ones with higher market value (Jones, 1999).

Five quantitative morphological traits (seed height, seed width, seed flatness, seed elongation and number of locules per pod) proved to be sufficient for discriminating true-type groups of accessions. The discriminant model, built on these five traits, was validated with 86.5% of accessions correctly assigned to the corresponding true-type group, previously classified based on genetic structure and phaseolin type analysis. This procedure was applied only for true-type groups, since accessions classified as offtypes possess intermediate forms between true-type groups that could bias the discriminant function. The discriminant analysis was subsequently applied to all the collection, and the offtype accessions were positioned among the true-type groups, as expected.

Isolation by distance analysis showed that there is no correlation between the geographic distance and the genetic differentiation among accessions. This absence of correlation may be due to an extensive gene flow resulting from traditional seed exchange practices such as trade at local markets or between farmers' family and neighbors. Those practices led to a maintenance of common bean genetic diversity in Portugal, denoted by the presence of almost all identified true-type groups in each region of the country.

On the other hand, we observed that the larger proportion of accessions came from the north central and north interior regions (39 and 35%, respectively). This fact is expected since those are the regions with a more suitable climate to cultivate bean, with higher values of annual precipitation associated with warmer summers, reducing the need for additional costly irrigation.

In order to take full advantage of this valuable germplasm, it is extremely important to complement this initial molecular and morphological characterization with more detailed phenotypic evaluations. This will allow the identification of accessions with increased breeding value; for instance, those more adapted to biotic and abiotic stresses, or possessing particular consumer-oriented quality traits. Yet, this is a time-consuming task in an extended collection such as this. To better exploit this germplasm collection, we developed smaller-size core collections representative of all the diversity from the original collection, with no redundancy. To select the accessions member of the core collection, we combined morphological diversity data (growth habit, seed shape, seed coat color, seed coat pattern, and seed size) with molecular marker data. Additionally, Shannon's diversity index was used to evaluate if there were significant differences in the morphological diversity of different core collection sizes. With this combined approach, we had the advantage of using not only simple easy-to-score phenotypic or passport data, normally more prone to missing data and environmental interaction dependence, but also DNA markers that can more accurately represent the genetic relationships and diversity of the entire collection (Díez et al., 2012). By employing the advanced maximization strategy (Schoen and Brown, 1993) we selected 37 accessions (25% of the entire collection analyzed), covering all alleles and morphological trait states present in the entire original collection. This percentage is within the 10–30% initial number of accessions generally kept in core collections (Wang et al., 2014). The smaller core sets (≤20 accessions) developed, although retaining with no significant differences the morphological trait states of the initial collection, had significant different allelic diversity. However, we should take into consideration that if a more detailed phenotypic evaluation (more traits evaluated) had taken place, the differences between the molecular and the morphological diversity of this collection might have been smaller. This highlighted the requirement of phenotyping the collection for other agronomic traits for specific breeding purposes. It is possible that new accessions might be added or removed as new data is acquired. Core collections, along with the germplasm that they represent, should be regarded as a dynamic concept (Díez et al., 2012). We observed a larger proportion of accessions from the Mesoamerican gene pool in the core set of 37 accessions as compared to that of the entire collection, which could be explained by the fact that the accessions of the AP1 type (Mesoamerican) had significantly higher allelic richness than both the B1P3 and B2P2 types (Andean). Additionally, 11 accessions out of the 37 (30%) were offtype accessions (composite, hybrids and non-corresponding) that, as it was proven, contain novel and relevant allelic information. The intermediate nature of some of these accessions opens new opportunities for using this unexplored material in common bean breeding.

Furthermore, the developed core collections, containing high genetic and morphological diversity, are potentially useful for conducting more detailed phenotypic assessments to deepen the characterization of this germplasm and speed up its use in breeding. This is especially important for traits highly influenced by the environment, such as yield potential. In the present study, since data was collected from one single environment, we limited the characterization mainly to highly heritable morphological traits that generally display low genotype by environment interaction (Debouck and Hidalgo, 1986; Pessarakli, 2001). Low heritable traits will be evaluated in the future using the developed core collection. Its smaller size will be more suitable for a multi-environmental evaluation needed to precisely characterize important agronomic traits such as yield. Also, climatic data and soil characteristics, which differ along the country, will be included in future studies in a more detailed multivariate analysis.

Moreover, the Portuguese core collection developed in this work may now integrate larger international bean collections to deepen the coverage of the worldwide diversity of this important crop. This could be of extreme importance to enrich international breeding or genetic programs and overcome several constraints affecting bean production in different parts of the world. Representative core collections have already proved to be useful tools for conservation purposes and to study resistance to abiotic and biotic stresses or other agronomic traits in several crop collections, such as pea (Grünwald et al., 2003), wheat (Shu et al., 2008), peanut (Chenault Chamberlin et al., 2010), and rice (Wang et al., 2010).

Finally, with this study, the complexity of the Portuguese bean germplasm, reflected by its genetic structure, was revealed. This knowledge is of vital importance for future genetic studies, using this underused but promising germplasm, such as association mapping studies. Those genome-wide studies might contribute to uncovering the genetic basis of several interesting traits and develop molecular tools to assist the selection of bean accessions with improved quality and agronomic performance.

## Author contributions

STL and MD performed the DNA isolation and the SSR genotyping. STL participated in the seed morphological characterization, data analysis, and drafted the manuscript. MMV coordinated the field experiment and performed the plant agronomic traits evaluations. ZS performed the statistical analysis of the molecular and phenotypic data and revised the manuscript critically. MCVP designed and coordinated the study and participated in the drafting and revising of the manuscript. All authors read and approved the final manuscript.

### Conflict of interest statement

The authors declare that the research was conducted in the absence of any commercial or financial relationships that could be construed as a potential conflict of interest.

## Footnotes

^1^http://www.fao.org/faostat

## References

[B1] Angioi S. A. Rau D. Attene G. Nanni L. Bellucci E. Logozzo G.. (2010). Beans in Europe: origin and structure of the European landraces of *Phaseolus vulgaris* L. Theor. Appl. Genet. 121, 829–843. 10.1007/s00122-010-1353-2 20490446

[B2] Angioi S. A. Rau D. Nanni L. Bellucci E. Papa R. Attene G. (2011). The genetic make-up of the European landraces of the common bean. Plant Genet. Resour. 9, 197–201. 10.1017/S1479262111000190

[B3] Bitocchi E. Bellucci E. Giardini A. Rau D. Rodriguez M. Biagetti E.. (2013). Molecular analysis of the parallel domestication of the common bean (*Phaseolus vulgaris*) in Mesoamerica and the Andes. New Phytol. 197, 300–313. 10.1111/j.1469-8137.2012.04377.x 23126683

[B4] Blair M. W. Díaz J. M. Hidalgo R. Díaz L. M. Duque M. C. (2007). Microsatellite characterization of Andean races of common bean (*Phaseolus vulgaris* L.). Theor. Appl. Genet. 116, 29–43. 10.1007/s00122-007-0644-8 17924092

[B5] Blair M. W. Hurtado N. Sharma P. (2012). New gene-derived simple sequence repeat markers for common bean (*Phaseolus vulgaris* L.). Mol. Ecol. Resour. 12, 661–668. 10.1111/j.1755-0998.2012.03136.x 22540633

[B6] Blair M. W. Pedraza F. Buendia H. F. Gaitán-Solís E. Beebe S. E. Gepts P.. (2003). Development of a genome-wide anchored microsatellite map for common bean (*Phaseolus vulgaris* L.). Theor. Appl. Genet. 107, 1362–1374. 10.1007/s00122-003-1398-6 14504741

[B7] Bowcock A. M. Ruiz-Linares A. Tomfohrde J. Minch E. Kidd J. R. Cavalli-Sforza L. L. (1994). High resolution of human evolutionary trees with polymorphic microsatellites. Nature 368, 455–457. 10.1038/368455a0 7510853

[B8] Burle M. L. Fonseca J. R. Kami J. A. Gepts P. (2010). Microsatellite diversity and genetic structure among common bean (*Phaseolus vulgaris* L.) landraces in Brazil, a secondary center of diversity. Theor. Appl. Genet. 121, 801–813. 10.1007/s00122-010-1350-5 20502861 PMC2940433

[B9] Câmara C. Urrea C. Schlegel V. (2013). Pinto beans (*Phaseolus vulgaris* L.) as a functional food: implications on human health. Agriculture 3, 90–111. 10.3390/agriculture3010090

[B10] Cavalli-Sforza L. L. Edwards A. W. F. (1967). Phylogenetic analysis: models and estimation procedures. Am. J. Hum. Genet. 19, 233–257. 10.1111/j.1558-5646.1967.tb03411.x 6026583 PMC1706274

[B11] Chenault Chamberlin K. D. Melouk H. A. Payton M. E. (2010). Evaluation of the U.S. peanut mini core collection using a molecular marker for resistance to Sclerotinia minor Jagger. Euphytica 172, 109–115. 10.1007/s10681-009-0065-7

[B12] Coelho R. C. Faria M. A. Rocha J. Reis A. Oliveira M. B. P. P. Nunes E. (2009). Assessing genetic variability in germplasm of *Phaseolus vulgaris* L. collected in Northern Portugal. Sci. Horticult. 122, 333–338. 10.1016/j.scienta.2009.05.035

[B13] Debouck D. G. Hidalgo R. (1986). Morphology of the Common Bean Plant. Phaseolus vulgaris. Cali: Centro Internacional de Agricultura Tropical (CIAT).

[B14] De Ron A. M. (2015). Grain legumes, in Handbook of Plant Breeding, ed De Ron A. M. (New York, NY: Springer-Verlag), 1–36. 10.1007/978-1-4939-2797-5

[B15] Díez C. M. Imperato A. Rallo L. Barranco D. Trujillo I. (2012). Worldwide core collection of olive cultivars based on simple sequence repeat and morphological markers. Crop Sci. 52, 211–221. 10.2135/cropsci2011.02.0110

[B16] Evanno G. Regnaut S. Goudet J. (2005). Detecting the number of clusters of individuals using the software structure: a simulation study. Mol. Ecol. 14, 2611–2620. 10.1111/j.1365-294X.2005.02553.x 15969739

[B17] Excoffier L. Laval G. Schneider S. (2005). Arlequin ver. 3.0: An integrated software package for population genetics data analysis. Evol. Bioinform. Online 1, 47–50. PMC2658868 19325852

[B18] Felsenstein J. (1985). Confidence limits on phylogenies: an approach using the bootstrap. Evolution 39, 783–791. 10.1111/j.1558-5646.1985.tb00420.x 28561359

[B19] Felsenstein J. (2004). PHYLIP (Phylogeny Inference Package), version 3.6. Seattle, WA: Distributed by the author, Department of Genomic Sciences, University of Washington.

[B20] Freitas G. Ganança J. F. T. Nóbrega H. Nunes E. Costa G. Slaski J. J. (2010). Morphological evaluation of common bean diversity on the Island of Madeira. Genet. Resour. Crop Evol. 58, 861–874. 10.1007/s10722-010-9624-y

[B21] Gaitan-Solis E. Duque M. C. Edwards K. J. Tohme J. (2002). Microsatellite repeats in common bean (*Phaseolus vulgaris*): isolation, characterization, and cross-species amplification in *Phaseolus* ssp. Crop Sci. 42, 2128–2136. 10.2135/cropsci2002.2128

[B22] Geffroy V. Sicard D. de Oliveira J. C. F. Sévignac M. Cohen S. Gepts P.. (1999). Identification of an ancestral resistance gene cluster involved in the coevolution process between *Phaseolus vulgaris* and its ffungal pathogen *Colletotrichum lindemuthianum*. Mol. Plant Microbe Interact. 12, 774–784. 10.1094/MPMI.1999.12.9.774 10494630

[B23] Gepts P. Bliss F. A. (1988). Dissemination pathways of common bean (*Phaseolus vulgaris, Fabaceae*) deduced from phaseolin electrophoretic variability. II Europe and Africa. Econ. Bot. 42, 86–104. 10.1007/BF02859038

[B24] Gepts P. Osborn T. C. Rashka K. Bliss F. A. (1986). Phaseolin-protein variability in wild forms and landraces of the common bean (*Phaseolus vulgaris*): evidence for multiple centers of domestication. Econ. Bot. 40, 451–468. 10.1007/BF02859659

[B25] Gil J. Ron A. D. (1992). Variation in *Phaseolus vulgaris* in the Northwest of the Iberian Peninsula. Plant Breed. 109, 313–319. 10.1111/j.1439-0523.1992.tb00190.x

[B26] Gioia T. Logozzo G. Attene G. Bellucci E. Benedettelli S. Negri V.. (2013). Evidence for introduction bottleneck and extensive inter-gene pool (Mesoamerica x Andes) hybridization in the European common bean (*Phaseolus vulgaris* L.) germplasm. PLoS ONE 8:e75974. 10.1371/journal.pone.0075974 24098412 PMC3788063

[B27] González A. M. Rodiño A. P. Santalla M. De Ron A. M. (2009). Genetics of intra-gene pool and inter-gene pool hybridization for seed traits in common bean (*Phaseolus vulgaris* L.) germplasm from Europe. Field Crops Res. 112, 66–76. 10.1016/j.fcr.2009.02.003

[B28] Goudet J. (1995). FSTAT (vers. 1.2): a computer program to calculate F-statistics. J. Hered. 86, 485–486. 10.1093/oxfordjournals.jhered.a111627

[B29] Goudet J. (2002). FSTAT. A Program for Windows to Estimate and Test Gene Diversities and Fixation Indices. Version 2.9.3. Edn.

[B30] Gouesnard B. Bataillon T. M. Decoux G. Rozale C. Schoen D. J. David J. L. (2001). MSTRAT: an algorithm for building germplasm core collections by maximizing allelic or phenotypic richness. J. Hered. 92, 93–94. 10.1093/jhered/92.1.93 11336240

[B31] Grünwald N. J. Coffman V. A. Kraft J. M. (2003). Sources of partial resistance to fusarium root rot in the *Pisum* core collection. Plant Dis. 87, 1197–1200. 10.1094/PDIS.2003.87.10.1197 30812722

[B32] Guzman P. Gilbertson R. L. Nodari R. Johnson W. C. Temple S. R. Mandala D. (1995). Characterization of variability in the fungus phaeoisariopsis-griseola suggests coevolution with the common bean (*Phaseolus vulgaris*). Phytopathology 85, 600–607. 10.1094/Phyto-85-600

[B33] IBPGR (1982). Phaseolus vulgaris Descriptors. Rome AGPG:IBPGR/81/1.

[B34] Jones A. L. (1999). Phaseolus bean: post-harvest operations, in Post-harvest Compendium, eds AGSI/FAO Mejia D. (Rome: Centro Internacional de Agricultura Tropical, FAO), 1–24.

[B35] Kalinowski S. T. (2004). Counting alleles with rarefaction: private alleles and hierarchical sampling designs. Conserv. Genet. 5, 539–543. 10.1023/B:COGE.0000041021.91777.1a

[B36] Kalinowski S. T. (2005). hp-rare 1.0: a computer program for performing rarefaction on measures of allelic richness. Mol. Ecol. Notes 5, 187–189. 10.1111/j.1471-8286.2004.00845.x

[B37] Kami J. Velasquez V. B. Debouck D. G. Gepts P. (1995). Identification of presumed ancestral DNA sequences of phaseolin in *Phaseolus vulgaris*. Proc. Natl. Acad. Sci. U.S.A. 92, 1101–1104. 10.1073/pnas.92.4.1101 7862642 PMC42645

[B38] Kelly J. D. Kolkman J. M. Schneider K. (1998). Breeding for yield in dry bean (*Phaseolus vulgaris* L.). Euphytica 102, 343–356. 10.1023/A:1018392901978

[B39] Kim K.-W. Chung H.-K. Cho G.-T. Ma K.-H. Chandrabalan D. Gwag J.-G.. (2007). PowerCore: a program applying the advanced M strategy with a heuristic search for establishing core sets. Bioinformatics 23, 2155–2162. 10.1093/bioinformatics/btm313 17586551

[B40] Koinange E. M. K. Singh S. P. Gepts P. (1996). Genetic control of the domestication syndrome in common bean. Crop Sci. 36, 1037–1045. 10.2135/cropsci1996.0011183X003600040037x

[B41] Kwak M. Gepts P. (2009). Structure of genetic diversity in the two major gene pools of common bean (*Phaseolus vulgaris* L., *Fabaceae*). Theor. Appl. Genet. 118, 979–992. 10.1007/s00122-008-0955-4 19130029

[B42] Kwak M. Toro O. Debouck D. G. Gepts P. (2012). Multiple origins of the determinate growth habit in domesticated common bean (*Phaseolus vulgaris*). Ann. Bot. 110, 1573–1580. 10.1093/aob/mcs207 23019270 PMC3503494

[B43] Leitão S. T. Almeida N. F. Moral A. Rubiales D. Vaz Patto M. C. (2013). Identification of resistance to rust (*Uromyces appendiculatus*) and powdery mildew (*Erysiphe diffusa)* in Portuguese common bean germplasm. Plant Breed. 132, 654–657. 10.1111/pbr.12094

[B44] Lewontin R. C. (1972). The apportionment of human diversity. Evol. Biol. 6, 381–398. 10.1007/978-1-4684-9063-3_14

[B45] Liu K. J. Muse S. V. (2005). PowerMarker: an integrated analysis environment for genetic marker analysis. Bioinformatics 21, 2128–2129. 10.1093/bioinformatics/bti282 15705655

[B46] Logozzo G. Donnoli R. Macaluso L. Papa R. Knüpffer H. Zeuli P. S. (2007). Analysis of the contribution of Mesoamerican and Andean gene pools to European common bean (*Phaseolus vulgaris* L.) germplasm and strategies to establish a core collection. Genet. Resour. Crop Evol. 54, 1763–1779. 10.1007/s10722-006-9185-2

[B47] Martins S. R. Vences F. J. Sáenz de Miera L. E. Barroso M. R. Carnide V. (2006). RAPD analysis of genetic diversity among and within Portuguese landraces of common white bean (*Phaseolus vulgaris* L.). Sci. Hortic. 108, 133–142. 10.1016/j.scienta.2006.01.031

[B48] Miklas P. N. Kelly J. D. Beebe S. E. Blair M. W. (2006). Common bean breeding for resistance against biotic and abiotic stresses: from classical to MAS breeding. Euphytica 147, 105–131. 10.1007/s10681-006-4600-5

[B49] Minch E. Ruiz-Linares A. Goldstein D. Feldman M. Cavalli-Sforza L. L. (1997). MICROSAT: A Computer Program for Calculating Various Statistics on Microsatellite Allele Data. v1.5d Edn. Stanford, CA: Stanford University.

[B50] Papa R. Bellucci E. Rossi M. Leonardi S. Rau D. Gepts P.. (2007). Tagging the signatures of domestication in common bean (*Phaseolus vulgaris*) by means of pooled DNA samples. Ann. Bot. 100, 1039–1051. 10.1093/aob/mcm151 17673468 PMC2759209

[B51] Papa R. Nanni L. Sicard D. Rau D. Attene G. (eds.). (2006). The evolution of genetic diversity in Phaseolus vulgaris L. New York, NY: Columbia University Press, 121–142. 10.7312/motl13316-007

[B52] Peakall R. O. D. Smouse P. E. (2006). genalex 6: genetic analysis in Excel. Population genetic software for teaching and research. Mol. Ecol. Notes 6, 288–295. 10.1111/j.1471-8286.2005.01155.x PMC3463245 22820204

[B53] Pessarakli M. (2001). Handbook of Plant and Crop Physiology. New York, NY: CRC Press 10.1201/9780203908426

[B54] Petry N. Boy E. Wirth J. Hurrell R. (2015). Review: The potential of the common bean (*Phaseolus vulgaris*) as a vehicle for iron biofortification. Nutrients 7, 1144–1173. 10.3390/nu7021144 25679229 PMC4344581

[B55] Pritchard J. K. Stephens M. Donnelly P. (2000). Inference of population structure using multilocus genotype data. Genetics 155, 945–959. 10835412 10.1093/genetics/155.2.945 PMC1461096

[B56] Raggi L. Tiranti B. Negri V. (2013). Italian common bean landraces: diversity and population structure. Genet. Resour. Crop Evol. 60, 1515–1530. 10.1007/s10722-012-9939-y

[B57] Rodiño A. P. Santalla M. De Ron A. M. Singh S. P. (2003). A core collection of common bean from the Iberian peninsula. Euphytica 131, 165–175. 10.1023/a:1023973309788

[B58] Rodiño A. P. Santalla M. González A. M. De Ron A. M. Singh S. P. (2006). Novel genetic variation in common bean from the Iberian Peninsula. Crop Sci. 46, 2540–2546. 10.2135/cropsci2006.02.0104

[B59] Rodiño A. P. Santalla M. Montero I. Casquero P. A. De Ron A. M. (2001). Diversity of common bean (*Phaseolus vulgaris* L.) germplasm from Portugal. Genet. Resour. Crop Evol. 48, 409–417. 10.1023/a:1012248002436

[B60] Rohlf F. (1997). NTSYS-PC 2.02i, Numerical Taxonomy and Multivariate Analysis System. Setauket, NY: Exeter Software.

[B61] Rohlf F. (2005). NTSYS-pc: Numerical Taxonomy and Multivariate Analysis System, ed S. E. Software version 2.2 Edn.

[B62] Rousset F. (1997). Genetic differentiation and estimation of gene flow from F-statistics under isolation by distance. Genetics 145, 1219–1228. 9093870 10.1093/genetics/145.4.1219 PMC1207888

[B63] Rousset F. (2008). GENEPOP'007: a complete re-implementation of the genepop software for Windows and Linux. Mol. Ecol. Resour. 8, 103–106. 10.1111/j.1471-8286.2007.01931.x 21585727

[B64] Santalla M. Rodiño A. P. Ron A. D. (2002). Allozyme evidence supporting southwestern Europe as a secondary center of genetic diversity for the common bean. Theor. Appl. Genet. 104, 934–944. 10.1007/s00122-001-0844-6 12582598

[B65] SAS/STAT® Software (2004). SAS/STAT® 9.1 User's Guide. Cary, NC: SAS Institute Inc.

[B66] Schoen D. J. Brown A. H. (1993). Conservation of allelic richness in wild crop relatives is aided by assessment of genetic markers. Proc. Natl. Acad. Sci. U.S.A. 90, 10623–10627. 10.1073/pnas.90.22.10623 8248153 PMC47829

[B67] Schuelke M. (2000). An economic method for the fluorescent labeling of PCR fragments. Nat. Biotechnol. 18, 233–234. 10.1038/72708 10657137

[B68] Shu K. Luo P. G. Zhang H. Y. Yan B. J. Zhang J. Zhang H. Q. (2008). Evaluation of leaf physiological and biochemical traits during senescence of the wheat core collection in the southwest of China. Can. J. Plant Sci. 88, 331–337. 10.4141/CJPS06018

[B69] Sicard D. Nanni L. Porfiri O. Bulfon D. Papa R. (2005). Genetic diversity of *Phaseolus vulgaris* L. and *P. coccineus* L. landraces in central Italy. Plant Breed. 124, 464–472. 10.1111/j.1439-0523.2005.01137.x

[B70] Singh S. P. Gepts P. Debouck D. G. (1991). Races of common bean (*Phaseolus vulgaris*, Fabaceae). Econ. Bot. 45, 379–396. 10.1007/BF02887079

[B71] Singh S. P. Molina A. Urrea C. A. Gutiérrez J. A. (1993). Use of interracial hybridization in breeding the race Durango common bean. Can. J. Plant Sci. 73, 785–793. 10.4141/cjps93-101

[B72] Torres A. M. Weeden N. F. Martin A. (1993). Linkage among isozyme, RFLP and RAPD markers in *Vicia faba*. Theor. Appl. Genet. 85, 937–945. 10.1007/BF00215032 24196143

[B73] Vaz Patto M. C. Moreira P. M. Carvalho V. Pego S. (2007). Collecting maize (*Zea mays* L. convar. mays) with potential technological ability for bread making in Portugal. Genet. Resour. Crop Evol. 54, 1555–1563. 10.1007/s10722-006-9168-3

[B74] Voysest O. Dessert M. (1991). Bean cultivars: classes and commercial seed types, in Common Beans. Research for Crop Improvement, eds van Schoonhoven A. Voysest O. (Cali: CAB International in association with Centro Internacional de Agricultura Tropical (CIAT)), 119–159.

[B75] Wang J. Guan Y. Wang Y. Zhu L. Wang Q. Hu Q.. (2014). A strategy for finding the optimal scale of plant core collection based on monte carlo simulation. Sci. World J. 2014:503473. 10.1155/2014/503473 24574893 PMC3918405

[B76] Wang X. Fjellstrom R. Jia Y. Yan W. G. Jia M. H. Scheffler B. E. (2010). Characterization of Pi-ta blast resistance gene in an international rice core collection. Plant Breed. 129, 491–501. 10.1111/j.1439-0523.2009.01706.x

[B77] White J. W. González A. (1990). Characterization of the negative association between seed yield and seed size among genotypes of common bean. Field Crops Res. 23, 159–175. 10.1016/0378-4290(90)90052-D

[B78] Yu K. Park S. J. Poysa V. Gepts P. (2000). Integration of simple sequence repeat (SSR) markers into a molecular linkage map of common bean (*Phaseolus vulgaris* L.). J. Hered. 91, 429–434. 10.1093/jhered/91.6.429 11218079

