# Supplementary material for: Establishing the Bases for Introducing the Unexplored Portuguese Common Bean Germplasm into the Breeding World
Source: Front Plant Sci. 2017 Jul 26;8:1296. doi: 10.3389/fpls.2017.01296 (PMC5526916; doi:10.3389/fpls.2017.01296)
Supplement: Supplementary file 1 [file Table1.PDF]

Supplementary Material

Establishing the bases for introducing the unexplored Portuguese common bean germplasm into the breeding world

Authors

Susana T. Leitão, Marco Dinis, Maria Manuela Veloso, Zlatko Šatović and Maria Carlota Vaz Patto*

Correspondence

*Corresponding author: cpatto@itqb.unl.pt

Table S1 – Passport information of the 175 common bean Portuguese accessions and 17 wild relatives and gene pool representatives evaluated in this study.

Accession	Region	Location	Latitude	Longitude	Altitude (m)
**0579**	northern interior	Bragança	4109N	0648W	460
**0583**	northern interior	Bragança	4109N	0648W	460
**0584**	northern interior	Bragança	4109N	0648W	460
**0587**	northern interior	Bragança	4132N	0657W	570
**0592**	northern interior	Bragança	4132N	0657W	570
**0600**	northern interior	Bragança	4120N	0643W	749
**0601**	northern interior	Bragança	4120N	0643W	749
**0602**	northern interior	Bragança	4132N	0657W	570
**0620**	northern interior	Bragança	4132N	0657W	570
**0621**	northern interior	Bragança	4132N	0657W	570
**0623**	northern interior	Bragança	4132N	0657W	n/a
**0632**	northern interior	Bragança	4120N	0643W	749
**0633**	northern interior	Bragança	4120N	0643W	749
**0635**	northern interior	Bragança	4120N	0643W	749
**0639**	northern interior	Bragança	4148N	0645W	673
**0642**	northern interior	Bragança	4129N	0616W	679
**0644**	northern interior	Bragança	4129N	0616W	679
**0645**	northern interior	Bragança	4129N	0616W	679
**0648**	northern interior	Bragança	4129N	0616W	679
**0654**	northern interior	Bragança	4148N	0645W	673
**0667**	northern interior	Bragança	4148N	0645W	673
**0670**	northern interior	Bragança	4148N	0645W	673
**0671**	northern interior	Bragança	4148N	0645W	673
**0675**	northern interior	Bragança	4148N	0645W	673
**0695**	northern interior	Bragança	4148N	0645W	673
**0698**	northern interior	Bragança	4145N	0630W	700
**0700**	northern interior	Bragança	4145N	0630W	700
**0706**	central north	Vila Real	4144N	0728W	368
**0735**	northern interior	Bragança	41508N	070005W	687
**0736**	northern interior	Bragança	41508N	070005W	687
**0737**	central north	Vila Real	4149N	0747W	990

**0747**	northern interior	Bragança	412924N	071039W	218
**0748**	northern interior	Bragança	412924N	071039W	218
**1631**	central south	Lisboa	3847N	0923W	180
**1636**	central south	Lisboa	3856N	0919W	242
**1644**	central south	Lisboa	3949N	0910W	21
**1651**	central south	Lisboa	3856N	0919W	242
**1653**	central south	Lisboa	3856N	0919W	242
**1654**	central south	Lisboa	3856N	0919W	242
**1662**	central south	Lisboa	3856N	0919W	242
**1663**	central south	Lisboa	3856N	0919W	242
**1867**	northern interior	Bragança	412924N	071039W	218
**1871**	northern interior	Bragança	412924N	071039W	218
**1877**	northern interior	Bragança	412924N	071039W	218
**1883**	northern interior	Bragança	412924N	071039W	218
**1884**	northern interior	Bragança	412924N	071039W	218
**1889**	northern interior	Bragança	4132N	0657W	570
**1892**	northern interior	Bragança	4132N	0657W	570
**1893**	northern interior	Bragança	4132N	0657W	n/a
**1897**	northern interior	Bragança	4120N	0643W	749
**1911**	northern interior	Bragança	4129N	0616W	679
**1917**	northern interior	Bragança	4129N	0616W	679
**1918**	northern interior	Bragança	4148N	0645W	673
**1926**	northern interior	Bragança	4148N	0645W	673
**1927**	northern interior	Bragança	4148N	0645W	673
**1932**	northern interior	Bragança	4148N	0645W	673
**1933**	northern interior	Bragança	4148N	0645W	673
**1937**	northern interior	Bragança	4148N	0645W	673
**1938**	northern interior	Bragança	4148N	0645W	673
**1943**	northern interior	Bragança	4148N	0645W	673
**1944**	northern interior	Bragança	4148N	0645W	673
**1948**	northern interior	Bragança	4148N	0645W	673
**1952**	northern interior	Bragança	4148N	0645W	673
**1955**	northern interior	Bragança	4132N	0657W	570
**1956**	northern interior	Bragança	4132N	0657W	570
**1961**	northern interior	Bragança	4132N	0657W	570
**1964**	northern interior	Bragança	4132N	0657W	570
**1966**	central north	Vila Real	4144N	0728W	368
**1975**	central north	Vila Real	4144N	0728W	368
**1976**	central north	Vila Real	4136N	0718W	425
**1979**	central north	Vila Real	4136N	0718W	425
**1984**	central north	Vila Real	4136N	0718W	425
**2081**	Madeira	Funchal	3240N	01704W	50
**2105**	Madeira	Funchal	n/a	n/a	n/a
**2126**	Madeira	Funchal	3245N	01649W	250
**2155**	Madeira	Funchal	3247N	01702W	150
**2159**	Madeira	Funchal	3249N	01706W	150
**2179**	Madeira	Funchal	3243N	01657W	690
**2189**	Madeira	Funchal	3243N	01701W	500

**2192**	Madeira	Funchal	3243N	01701W	500
**4038**	central north	Viseu	405376N	0074250W	862
**4039**	central north	Viseu	n/a	n/a	n/a
**4044**	central north	Viseu	405397N	0074339W	844
**4048**	central north	Guarda	405301N	74840W	774
**4049**	central north	Guarda	405301N	74840W	774
**4050**	central north	Guarda	405301N	74840W	774
**4051**	central north	Guarda	405301N	74840W	774
**4067**	central north	Viseu	405055N	0075624W	471
**4070**	central north	Viseu	405483N	75838W	502
**4071**	central north	Viseu	405483N	75838W	502
**4072**	central north	Viseu	405483N	75838W	502
**4073**	central north	Viseu	n/a	n/a	n/a
**4081**	central north	Viseu	405713N	75484W	867
**4085**	central north	Viseu	404181N	80497W	567
**4088**	central north	Viseu	404181N	80497W	567
**4097**	central north	Viseu	404181N	80497W	567
**4100**	central north	Viseu	403933N	80918W	747
**4108**	central north	Viseu	403804N	80311W	434
**4110**	central north	Viseu	4039N	0754W	475
**4112**	central north	Viseu	n/a	n/a	n/a
**4119**	central north	Viseu	4039N	0754W	475
**4120**	central north	Viseu	4039N	0754W	475
**4127**	central north	Guarda	404575N	073444W	609
**4133**	central north	Guarda	404505N	073217W	544
**4135**	central north	Guarda	404505N	073217W	544
**4139**	central north	Guarda	n/a	n/a	n/a
**4144**	central north	Guarda	405132N	0073019W	618
**4150**	central north	Guarda	401951N	0074118W	794
**4162**	central north	Guarda	403153N	0073423W	459
**4164**	central north	Guarda	403153N	0073423W	459
**4182**	central north	Guarda	404012N	0072470W	426
**4185**	central north	Coimbra	401992N	0075055W	269
**4189**	central north	Coimbra	401992N	0075055W	269
**4194**	central north	Coimbra	n/a	n/a	n/a
**4290**	central north	Guarda	4032N	0716W	900
**4295**	south	Faro	3718N	0848W	36
**4300**	south	Faro	3700N	0756W	9
**4306**	south	Faro	n/a	n/a	n/a
**5246**	central north	Viseu	n/a	n/a	n/a
**5248**	central north	Viseu	n/a	n/a	n/a
**5249**	central north	Viseu	4053192N	805983	453
**5285**	north coast	Braga	4130N	0759W	300
**5286**	north coast	Braga	4130N	0759W	300
**5287**	north coast	Braga	4130N	0759W	300
**5288**	central north	Aveiro	4038N	0839W	8
**5290**	Azores	Açores	3744N	2539W	48
**5291**	South	Faro	3700N	0756W	9

**5292**	South	Faro	3700N	0756W	9
**5293**	South	Faro	n/a	n/a	n/a
**5295**	South	Faro	3708N	0801W	171
**5296**	South	Faro	3718N	0848W	36
**5297**	South	Faro	3708N	0801W	171
**5298**	northern interior	Bragança	4109N	0648W	460
**5300**	northern interior	Bragança	4148N	0645W	673
**5302**	north coast	Braga	4116N	0816W	300
**5363**	central south	Lisboa	3856N	0919W	242
**5365**	central north	Guarda	4032N	0716W	540
**5366**	central north	Guarda	4032N	0716W	540
**5367**	central north	Guarda	4032N	0716W	540
**5368**	central north	Guarda	4032N	0716W	540
**5369**	central north	Aveiro	4038N	0839W	8
**5370**	north coast	Braga	4132N	0836W	34
**5371**	north coast	Braga	4132N	0836W	34
**5372**	central south	Leiria	3921N	0909W	51
**5376**	south	Faro	3700N	0756W	9
**5377**	south	Faro	3700N	0756W	9
**5378**	central south	Oeste	3856N	0919W	240
**5379**	northern interior	Bragança	4148N	0645W	673
**5380**	north coast	Braga	4132N	0836W	34
**5381**	north coast	Braga	4132N	0836W	34
**4085-B**	central north	Viseu	n/a	n/a	n/a
**4085-M**	central north	Viseu	n/a	n/a	n/a
**4149-R**	central north	Guarda	n/a	n/a	n/a
**4149-V**	central north	Guarda	401951N	0074118W	794
**4179-B**	central north	Guarda	n/a	n/a	n/a
**4179**	central north	Guarda	403902N	0072453W	441
**4182-C**	central north	Guarda	n/a	n/a	n/a
**4182-M**	central north	Guarda	n/a	n/a	n/a
**5382**	north coast	Braga	n/a	n/a	n/a
**5383**	north coast	Braga	n/a	n/a	n/a
**GC-17T**	central north	Coimbra	n/a	n/a	n/a
**5384**	central south	Santarém	n/a	n/a	n/a
**GC-34T**	central north	Guarda	n/a	n/a	n/a
**GC-35**	central north	Guarda	n/a	n/a	n/a
**5385**	central north	Guarda	n/a	n/a	n/a
**5386**	central north	Guarda	n/a	n/a	n/a
**GC-40**	central north	Guarda	n/a	n/a	n/a
**5387**	central north	Guarda	n/a	n/a	n/a
**5388**	central north	Viseu	n/a	n/a	n/a
**5389**	central north	Viseu	n/a	n/a	n/a
**5391**	south	Faro	n/a	n/a	n/a
**GC-51**	south	Faro	n/a	n/a	n/a
**GC-52**	south	Faro	n/a	n/a	n/a
**5392**	south	Faro	n/a	n/a	n/a
**Tarrestre**	north coast	Braga			

**Gene pool representatives and wild relatives (CIAT[1])**	**Country**	**Gene pool**	**Eco-geographic race**	**Seed Storage Proteins**	**Ecology**
**G11360**	Mexico	Mesoamerican	Jalisco	S	Cultivated landrace
**G12597**	Peru	Andean	Peru	n/a	Cultivated landrace
**G1525**	Chile	Andean	Chile	H1	Cultivated landrace
**G19833**	Peru	Andean	Peru	H1	Cultivated landrace
**G19895**	Argentina	wild		J3, J1, T	Wild
**G19908**	Guatemala	wild		M16, M10	Wild
**G2333**	Mexico	Mesoamerican	Guatemala	S	Cultivated landrace
**G23429**	Mexico	wild		M4, M17	Wild
**G23444**	Bolivia	wild		T, Ca	Wild
**G23589**	Peru	wild		H2	Wild
**G24404**	Colombia	wild		L (2D)	Wild
**G4474**	Chile	Andean	Chile	S, B	Cultivated landrace
**G4494**	Colombia	Andean	Nueva Granada	T	Cultivated commercial variety
**G51105**	Colombia	Mesoamerican	Mesoamerica	S	Cultivated commercial variety
**G51294**	Colombia	Mesoamerican	Mesoamerica	B	Cultivated commercial variety
**G685**	Guatemala	Mesoamerican	Guatemala	Sb, S	Cultivated commercial variety
**G9603**	Brazil	Andean	Nueva Granada	T	Cultivated landrace

[1] http://ciat.cgiar.org/what-we-do/crop-conservation-and-use/bean-diversity/
