# Supplementary material for: Establishing the Bases for Introducing the Unexplored Portuguese Common Bean Germplasm into the Breeding World
Source: Front Plant Sci. 2017 Jul 26;8:1296. doi: 10.3389/fpls.2017.01296 (PMC5526916; doi:10.3389/fpls.2017.01296)
Supplement: Supplementary file 2 [file Table2.PDF]

Supplementary Material

Establishing the bases for introducing the unexplored Portuguese common bean germplasm into the breeding world

Authors

Susana T. Leitão, Marco Dinis, Maria Manuela Veloso, Zlatko Šatović and Maria Carlota Vaz Patto*

Correspondence

*Corresponding author: cpatto@itqb.unl.pt

Table S2 – List of the 21 microsatellite markers used in this study.

Locus	Linkage group	Type	Reference
**BM143**	2	Non-gene derived	Gaitan-Solis et al. 2002
**BM146**	1	Non-gene derived	Gaitan-Solis et al. 2002
**BM151**	8	Non-gene derived	Gaitan-Solis et al. 2002
**BM157**	10	Non-gene derived	Gaitan-Solis et al. 2002
**BM172**	3	Non-gene derived	Gaitan-Solis et al. 2002
**BM188**	9	Non-gene derived	Gaitan-Solis et al. 2002
**BM197**	3	Non-gene derived	Gaitan-Solis et al. 2002
**BM210**	7	Non-gene derived	Gaitan-Solis et al. 2002
**BMD12**	6	Non-gene derived	Blair et al. 2003
**BMD20**	5	Gene derived	Blair et al. 2003
**BMD22**	11	Gene derived	Blair et al. 2003
**BMD25**	8	Gene derived	Blair et al. 2003
**BMD42**	10	Non-gene derived	Blair et al. 2003
**BMD45**	1	Gene derived	Blair et al. 2003
**BMD53**	5	Gene derived	Blair et al. 2003
**GATS91**	2	Non-gene derived	Gaitan-Solis et al. 2002

**PV-ag001**	11	Gene derived	Yu et al. 2000
**PV-ag003**	1	Gene derived	Yu et al. 2000
**PV-at007**	9	Gene derived	Yu et al. 2000
**PV-atcc003**	7	Gene derived	Yu et al. 2000
**PV-ctt001**	4	Gene derived	Yu et al. 2000
