# Supplementary material for: Establishing the Bases for Introducing the Unexplored Portuguese Common Bean Germplasm into the Breeding World
Source: Front Plant Sci. 2017 Jul 26;8:1296. doi: 10.3389/fpls.2017.01296 (PMC5526916; doi:10.3389/fpls.2017.01296)
Supplement: Supplementary file 3 [file Table3.PDF]

Supplementary Material

Establishing the bases for introducing the unexplored Portuguese common bean germplasm into the breeding world

Authors

Susana T. Leitão, Marco Dinis, Maria Manuela Veloso, Zlatko Šatović and Maria Carlota Vaz Patto*

Correspondence

*Corresponding author: cpatto@itqb.unl.pt

Table S3 - Allelic diversity and polymorphic information content of 21 microsatellite loci scored in 175 bean accessions from the Portuguese collection and in the 17 representatives of Andean and Mesoamerican races and wild relatives, held at CIAT seedbank (a total of 192 accessions and 1826 individuals).

		**Origin of accessions**								
**SSR Markers**		**Portuguese**			**Andean and Mesoamerican race representatives and wild relatives**			**All**		
**Name**	Repetitive sequence	Product size range (basepairs)	No. alleles	PIC	Product size range (basepairs)	No. alleles	PIC	Product size range (basepairs)	No. alleles	PIC
**BM143**	$(GA)_{35}$	131-205	20	0.763	133-191	20	0.914	131-205	26	0.791
**BM146**	$(CTGTTG)_4(CTG)_4$ $(TTG)_3(CTG)_3(CTG)_4$	292-307	4	0.137	292-304	4	0.149	292-307	5	0.139
**BM151**	$(CT)_{14}$	147-171	8	0.511	163-171	5	0.713	147-171	8	0.563
**BM157**	$(GA)_{16}$	117-155	11	0.711	115-207	10	0.735	115-207	16	0.729
**BM172**	$(GA)_{23}$	82-136	13	0.685	94-134	13	0.865	82-136	19	0.728
**BM188**	$(CA)_{18}(TA)_7$	172-210	15	0.697	172-210	13	0.879	172-210	16	0.736
**BM197**	$(GT)_8$	216-224	4	0.303	216-220	3	0.549	216-224	4	0.338
**BM210**	$(CT)_{15}$	177-205	12	0.634	179-213	10	0.840	177-213	16	0.663

**BMd12**	$(AGC)_7$	169-184	5	0.256	178-181	2	0.364	169-184	5	0.273
**BMd20**	$(TA)_5$	137-169	7	0.315	137-147	6	0.470	137-169	8	0.333
**BMd22**	$(TC)_6$	135-137	2	0.333	137-139	2	0.346	135-139	3	0.409
**BMd25**	$(GAT)_6$	126-135	4	0.356	132-135	2	0.207	126-135	4	0.367
**BMd42**	$(AT)_5$	141-191	11	0.694	145-181	8	0.746	141-191	13	0.717
**BMd45**	$(AG)_5$	107-187	8	0.303	109-147	2	0.362	107-187	8	0.314
**BMd53**	$(GTA)_5$	109-127	3	0.298	124-127	2	0.374	109-127	3	0.312
**GATS91**	$(GA)_{17}$	232-278	19	0.808	222-276	15	0.909	222-278	21	0.828
**PV-ag001**	$(GA)_{11}$	152-192	8	0.568	164-188	8	0.771	152-192	11	0.608
**PV-ag003**	$(AG)_8$	182-186	3	0.261	178-186	5	0.551	178-186	5	0.298
**PV-at007**	$(AT)_{12}$	207-247	21	0.820	203-239	13	0.863	203-247	22	0.832
**PV-atcc003**	$(ATCC)_3$	188-192	2	0.151	188-192	2	0.299	188-192	2	0.170
**PV-ctt001**	$(CTT)_3T_3(CTT)_6$	163-193	8	0.377	166-187	6	0.713	163-193	10	0.432
**Average**			8.95	0.475		7.19	0.601		10.71	0.504
**Total**			188			151			225	

SSR (simple-sequence repeat), PIC (polymorphic information content)
