# Supplementary material for: Establishing the Bases for Introducing the Unexplored Portuguese Common Bean Germplasm into the Breeding World
Source: Front Plant Sci. 2017 Jul 26;8:1296. doi: 10.3389/fpls.2017.01296 (PMC5526916; doi:10.3389/fpls.2017.01296)
Supplement: Supplementary file 4 [file Table4.PDF]

Supplementary Material

Establishing the bases for introducing the unexplored Portuguese common bean germplasm into the breeding world

Authors
Susana T. Leitão, Marco Dinis, Maria Manuela Veloso, Zlatko Šatović and Maria Carlota Vaz Patto*
Correspondence
*Corresponding author: cpatto@itqb.unl.pt

Table S4 - Genetic diversity of the two groups (Portuguese vs. Andean and Mesoamerican race representatives and wild bean relatives) on individual level across 21 microsatellite markers.

	**Origin of accessions**				
**Parameter**	**Portugal**		**Andean and Mesoamerican race representatives and wild relatives**		**P(ϕ)**[1]
	**Average**[2]	**Range**	**Average**	**Range**	
No. samples (individual plants)	1675		151		
Total number of alleles	188		151		
Number of alleles (N_a)	8.952	(2.00-21.00)	7.190	(2.00-20.00)	
Allelic richness (N_{ar})	6.763	(2.00-20.10)	7.180	(2.00-20.00)	0.479
Effective number of alleles (N_e)[3]	2.081	(1.17-6.01)	2.761	(1.21-14.95)	
Number of private alleles (N_{pr})	3.524	(0.00-9.00)	1.762	(0.00-6.00)	
Total number of private alleles per region	74		37		
Private allelic richness (N_{par})	1.818	(0.00-8.26)	2.235	(0.00-8.00)	
Observed heterozygosity (H_O)	0.027	(0.00-0.18)	0.066	(0.00-0.42)	0.026
Expected heterozygosity (H_E)	0.519	(0.15-0.83)	0.636	(0.17-0.93)	0.000

[1]Significance of repeated measures ANOVA
[2]Average (and range) across microsatellite markers
[3]Harmonic mean
