# Supplementary material for: Establishing the Bases for Introducing the Unexplored Portuguese Common Bean Germplasm into the Breeding World
Source: Front Plant Sci. 2017 Jul 26;8:1296. doi: 10.3389/fpls.2017.01296 (PMC5526916; doi:10.3389/fpls.2017.01296)
Supplement: Supplementary file 5 [file Table5.PDF]

Supplementary Material

Establishing the bases for introducing the unexplored Portuguese common bean germplasm into the breeding world

Authors

Susana T. Leitão, Marco Dinis, Maria Manuela Veloso, Zlatko Šatović and Maria Carlota Vaz Patto*

Correspondence

*Corresponding author: cpatto@itqb.unl.pt

Table S5 - Genetic diversity of Portuguese common bean accessions grouped by region of origin as assessed by 21 microsatellite loci.

Parameter	Region						P(KW)[1]
	north coast	northern interior	central north	central south	south	Madeira	
No. accessions	11	60	68	12	15	8	
Number of alleles (N_a)	1.372	1.381	1.317	1.349	1.225	1.339	
Allelic richness (N_{ar})	1.109	1.109	1.090	1.100	1.073	1.117	0.506
Effective number of alleles (N_e)[2]	1.125	1.126	1.104	1.116	1.084	1.140	
Number of private alleles (N_{pr})	0.273	0.317	0.191	0.167	0.133	0.000	
Total number of private alleles per region	4	25	17	4	2	3	
Private allelic richness (N_{par})	0.007	0.007	0.005	0.012	0.005	0.018	
Observed heterozygosity (H_O)	0.033	0.033	0.024	0.024	0.015	0.012	0.147
Expected heterozygosity (H_E)	0.112	0.112	0.094	0.104	0.078	0.123	0.476
Genetic distance (D_{Chord})[3]	0.237	0.199	0.180	0.209	0.175	0.157	

[1]Significance of Kruskal-Wallis test

[2]Harmonic mean

[3]Average pairwise Cavalli-Sforza and Edwards' chord distances among accessions
