# Supplementary material for: Establishing the Bases for Introducing the Unexplored Portuguese Common Bean Germplasm into the Breeding World
Source: Front Plant Sci. 2017 Jul 26;8:1296. doi: 10.3389/fpls.2017.01296 (PMC5526916; doi:10.3389/fpls.2017.01296)
Supplement: Supplementary file 6 [file Table6.PDF]

Supplementary Material

Establishing the bases for introducing the unexplored Portuguese common bean germplasm into the breeding world

Authors

Susana T. Leitão, Marco Dinis, Maria Manuela Veloso, Zlatko Šatović and Maria Carlota Vaz Patto*

Correspondence

*Corresponding author: cpatto@itqb.unl.pt

Table S6 - Phaseolin patterns of the 175 Portuguese bean accessions.

Gel bands profile pattern (fragments in base pairs)	No. PT accessions	Phaseolin haplotype (our classification)	CIAT's classification[1]	Gene pool of the phaseolin type
**268, 290**	33	P1	S (Sanilac)	Mesoamerican
**268, 283, 290, 304**	69	P2	H (Huevo de Huanchaco) or C (Contender)	Andean
**268, 283, 290*, 304**	66	P3	T (Tendergreen)	Andean
**Other fragment sizes**	7	nd[2]		

*indicates fragment with weaker signal than the other ones
[1] http://ciat.cgiar.org/what-we-do/crop-conservation-and-use/bean-diversity/
[2]nd – not defined
