# Supplementary material for: Establishing the Bases for Introducing the Unexplored Portuguese Common Bean Germplasm into the Breeding World
Source: Front Plant Sci. 2017 Jul 26;8:1296. doi: 10.3389/fpls.2017.01296 (PMC5526916; doi:10.3389/fpls.2017.01296)
Supplement: Supplementary file 7 [file Table7.PDF]

Supplementary Material

Establishing the bases for introducing the unexplored Portuguese common bean germplasm into the breeding world

Authors

Susana T. Leitão, Marco Dinis, Maria Manuela Veloso, Zlatko Šatović and Maria Carlota Vaz Patto*

Correspondence

*Corresponding author: cpatto@itqb.unl.pt

Table S7 - Morphological characterization of the Portuguese bean accessions. ANOVA's F-test among true type accessions, and Tukey's test between pairs of true type groups are shown.

		Bean true type groups				Bean offtype groups			All
		AP1	B1P3	B2P2	*P(ϕ)*	Composite	Hybrid	Non-corresponding	
**Seed length (mm)**	Range	8.58-17.00	11.9-17.88	9.89-15.93		9.03-16.18	10.69-18.91	11.09-18.15	8.58-18.91
	Mean	12.68	15.18	12.79	*0.0001*	11.980	15.103	13.894	13.646
	Tukey's test[1]	*b*	*a*	*b*					
**Seed width (mm)**	Range	4.56-6.19	5.27-6.84	5.35-8.12		5.13-6.87	4.8-7.64	5.16-7.75	4.56-8.12
	Mean	5.20	5.81	6.72	*0.0001*	6.074	6.185	6.394	6.199
	Tukey's test	*c*	*b*	*a*					
**Seed height (mm)**	Range	6.04-9.33	6.58-8.27	6.83-9.3		5.34-8.13	6.47-9.15	6.36-8.76	5.34-9.33
	Mean	7.43	7.45	8.03	*0.0003*	7.041	7.666	7.793	7.719
	Tukey's test	*b*	*b*	*a*					
**100-seeds weight (g)**	Range	19.1-60.71	35.56-65.69	27.97-69.21		22.52-66.06	26.87-71.5	32.5-68.94	19.1-71.5
	Mean	36.86	49.11	47.47	*0.0001*	36.888	52.088	48.982	46.714
	Tukey's test	*b*	*a*	*a*					

**Seeds per pod**	Range	3.06-7.71	2.73-4.92	2.71-7.13		3.15-5.7	3.3-5.4	2.73-6.05	2.71-7.71
	Mean	4.98	3.67	4.62	*0.0001*	4.600	4.084	4.308	4.383
	Tukey's test	*a*	*b*	*a*					
**Locules per pod**	Range	5-10.5	4-7	5-9		6-9	5-7.5	4-9	4-10.5
	Mean	7.40	5.58	6.83	*0.0001*	7.250	6.211	6.345	6.547
	Tukey's test	*a*	*b*	*a*					
**Elongation**	Range	1.38-2.12	1.6-2.36	1.23-2.13		1.37-2.14	1.4-2.52	1.35-2.41	1.23-2.52
	Mean	1.71	2.04	1.60	*0.0001*	1.710	1.978	1.800	1.780
	Tukey's test	*b*	*a*	*b*					
**Flatness**	Range	1.17-1.83	1.12-1.46	1.09-1.39		1.04-1.26	1.08-1.49	1.1-1.62	1.04-1.83
	Mean	1.43	1.29	1.20	*0.0001*	1.156	1.244	1.228	1.257
	Tukey's test	*a*	*b*	*c*					
**Flatness index**	Range	1.44-2.58	1.6-2.39	1.21-2.06		1.35-1.79	1.31-2.3	1.31-2.15	1.21-2.58
	Mean	1.94	1.95	1.57	*0.0001*	1.562	1.855	1.721	1.750
	Tukey's test	*a*	*a*	*b*					

[1]Different letters in Tukey's test means significantly differences between true type groups.
