# Supplementary material for: Establishing the Bases for Introducing the Unexplored Portuguese Common Bean Germplasm into the Breeding World
Source: Front Plant Sci. 2017 Jul 26;8:1296. doi: 10.3389/fpls.2017.01296 (PMC5526916; doi:10.3389/fpls.2017.01296)
Supplement: Supplementary file 8 [file Table8.PDF]

Supplementary Material

Establishing the bases for introducing the unexplored Portuguese common bean germplasm into the breeding world

Authors

Susana T. Leitão, Marco Dinis, Maria Manuela Veloso, Zlatko Šatović and Maria Carlota Vaz Patto*

Correspondence

*Corresponding author: cpatto@itqb.unl.pt

Table S8 - Presence or absence of seed coat pattern in the 150 Portuguese bean accessions. Significance of the likelihood-ratio chi-square is shown among true type groups.

		**Seed coat pattern**	
**Accession group type**	Subtype	Absent (plain seeds)	Present
**True type**	AP1	14	7
	B1P3	13	12
	B2P2	27	23
	P(χ)	*0.563*	
**Offtype**	Composite	5	1
	Hybrid	12	7
	Non-corresponding	19	10
	Total	90	60
