# Supplementary material for: Establishing the Bases for Introducing the Unexplored Portuguese Common Bean Germplasm into the Breeding World
Source: Front Plant Sci. 2017 Jul 26;8:1296. doi: 10.3389/fpls.2017.01296 (PMC5526916; doi:10.3389/fpls.2017.01296)
Supplement: Supplementary file 9 [file Table9.PDF]

Supplementary Material

Establishing the bases for introducing the unexplored Portuguese common bean germplasm into the breeding world

Authors

Susana T. Leitão, Marco Dinis, Maria Manuela Veloso, Zlatko Šatović and Maria Carlota Vaz Patto*

Correspondence

*Corresponding author: cpatto@itqb.unl.pt

Table S9 - Regions of origin of the 150 analyzed bean accessions distributed by accession group type. Significance of the likelihood-ratio chi-square among regions is shown for true types accession.

		**Region**[1]							
**Accession group type**	Subtype	1	2	3	4	5	6	7	Total
**True type**	AP1	4	8	4	3	1	0	1	21
	B1P3	3	5	12	1	3	0	1	25
	B2P2	3	22	13	2	5	1	4	50
	$P(\chi^2)$				*0.316*				
**Offtype**	Composite	0	4	2	0	0	0	0	6
	Hybrid	1	6	7	1	3	0	1	19
	Non-corresponding	0	13	12	4	0	0	0	29
	Total	11	58	50	11	12	1	7	150

[1]1 **-** north coast, 2 – northern interior, 3 – central north, 4 – central south, 5 – south, 6 – The Azores, 7 – Madeira
