# Supplementary material for: Establishing the Bases for Introducing the Unexplored Portuguese Common Bean Germplasm into the Breeding World
Source: Front Plant Sci. 2017 Jul 26;8:1296. doi: 10.3389/fpls.2017.01296 (PMC5526916; doi:10.3389/fpls.2017.01296)
Supplement: Supplementary file 10 [file Table10.PDF]

Supplementary Material

Establishing the bases for introducing the unexplored Portuguese common bean germplasm into the breeding world

Authors

Susana T. Leitão, Marco Dinis, Maria Manuela Veloso, Zlatko Šatović and Maria Carlota Vaz Patto*

Correspondence

*Corresponding author: cpatto@itqb.unl.pt

Table S10 - Pearson's correlation coefficients (r) between pairs of morphological quantitative traits (below diagonal). Above diagonal the P-value test of significance is shown.

Trait	Seed length	Seed width	Seed height	100-seed weight	No. of seeds per pod	Locules per pod	Elongation (L/H)	Flatness (H/W)	Flatness index = (L+H)/2W
**Seed length**		ns	ns	***	***	***	***	***	***
**Seed width**	-0.136		***	***	*	*	***	***	***
**Seed height**	0.140	0.659		***	***	**	***	ns	*
**100-seed weight**	0.706	0.470	0.627		***	***	***	ns	**
**No. of seeds per pod**	-0.337	-0.204	-0.322	-0.376		***	ns	ns	ns
**Locules per pod**	-0.365	-0.187	-0.260	-0.418	0.773		*	ns	ns
**Elongation (L/H)**	0.841	-0.458	-0.410	0.317	-0.137	-0.193		ns	***
**Flatness (H/W)**	0.281	-0.632	0.159	-0.003	-0.061	-0.014	0.147		***
**Flatness index = (L+H)/2W**	0.759	-0.704	-0.161	0.225	-0.140	-0.155	0.771	0.740	

P-value significant level: * 0.01 < p value < 0.05, **0.001 <p value <0.01, *** p value < 0.001
ns: not significant (p value > 0.05)
