# Supplementary material for: Establishing the Bases for Introducing the Unexplored Portuguese Common Bean Germplasm into the Breeding World
Source: Front Plant Sci. 2017 Jul 26;8:1296. doi: 10.3389/fpls.2017.01296 (PMC5526916; doi:10.3389/fpls.2017.01296)
Supplement: Supplementary file 14 [file Image3.PDF]

Supplementary Material

Establishing the bases for introducing the unexplored Portuguese common bean germplasm into the breeding world

Authors

Susana T. Leitão, Marco Dinis, Maria Manuela Veloso, Zlatko Šatović and Maria Carlota Vaz Patto*

Correspondence

*Corresponding author: cpatto@itqb.unl.pt

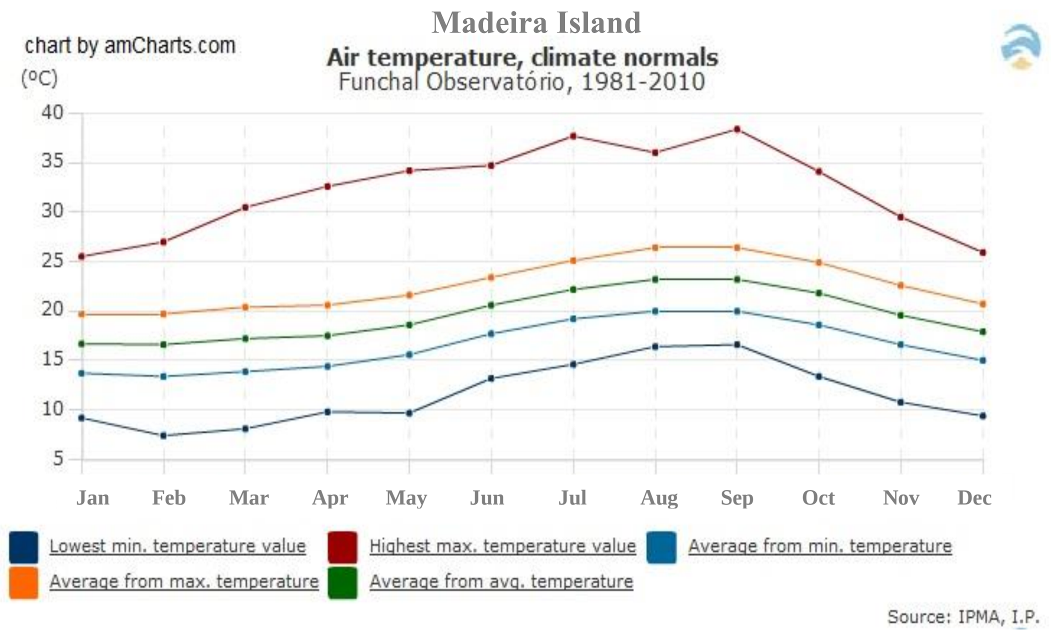

A

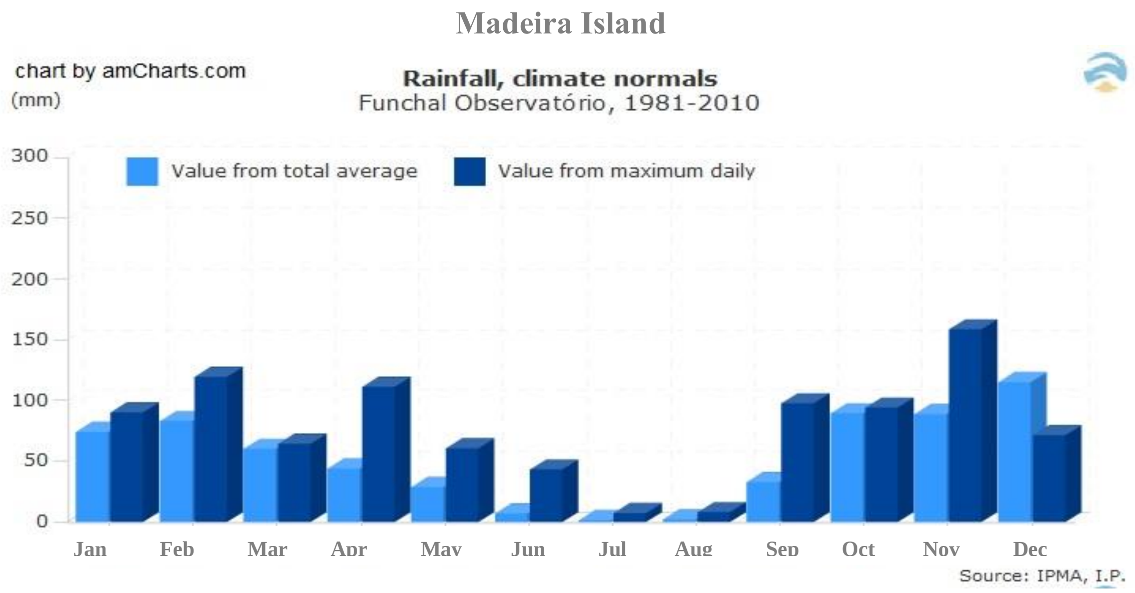

B

S. Miguel Island – The Azores

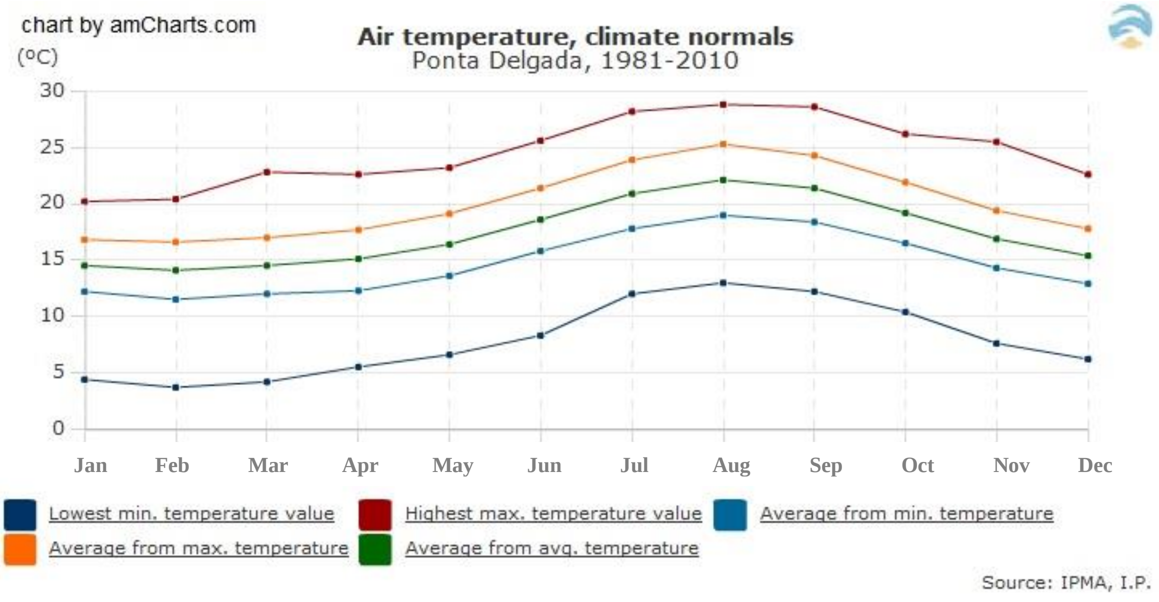

C

S. Miguel Island – The Azores

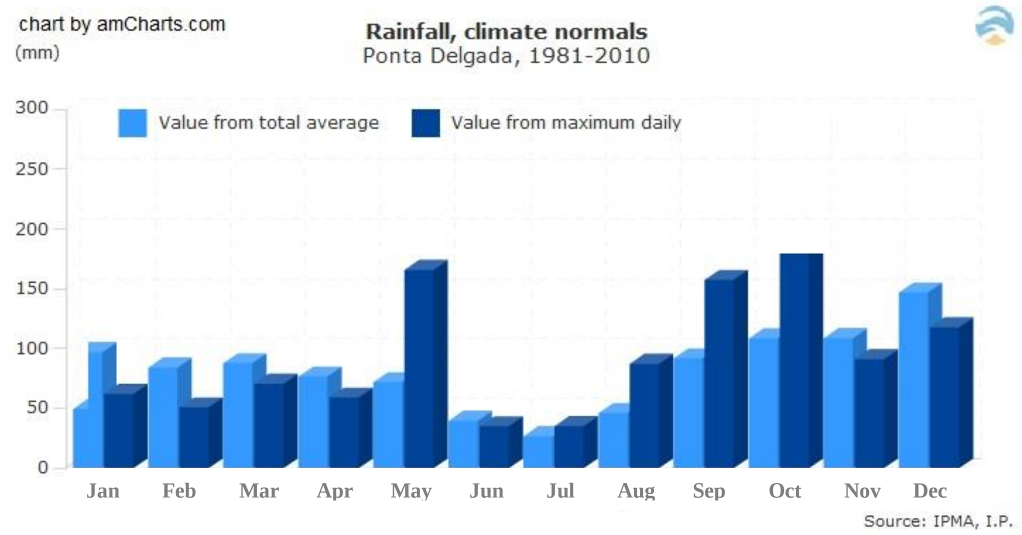

D

Supplementary Figure 3: Portuguese Autonomous Regions weather maps: Air temperature (ºC) and rainfall (mm). A and B – Madeira Island; C and D – S. Miguel Island (The Azores) (adapted from www.ipma.pt).
