# Supplementary material for: Establishing the Bases for Introducing the Unexplored Portuguese Common Bean Germplasm into the Breeding World
Source: Front Plant Sci. 2017 Jul 26;8:1296. doi: 10.3389/fpls.2017.01296 (PMC5526916; doi:10.3389/fpls.2017.01296)
Supplement: Supplementary file 15 [file Image4.PDF]

Supplementary Material

Establishing the bases for introducing the unexplored Portuguese common bean germplasm into the breeding world

Authors

Susana T. Leitão, Marco Dinis, Maria Manuela Veloso, Zlatko Šatović and Maria Carlota Vaz Patto*

Correspondence

*Corresponding author: cpatto@itqb.unl.pt

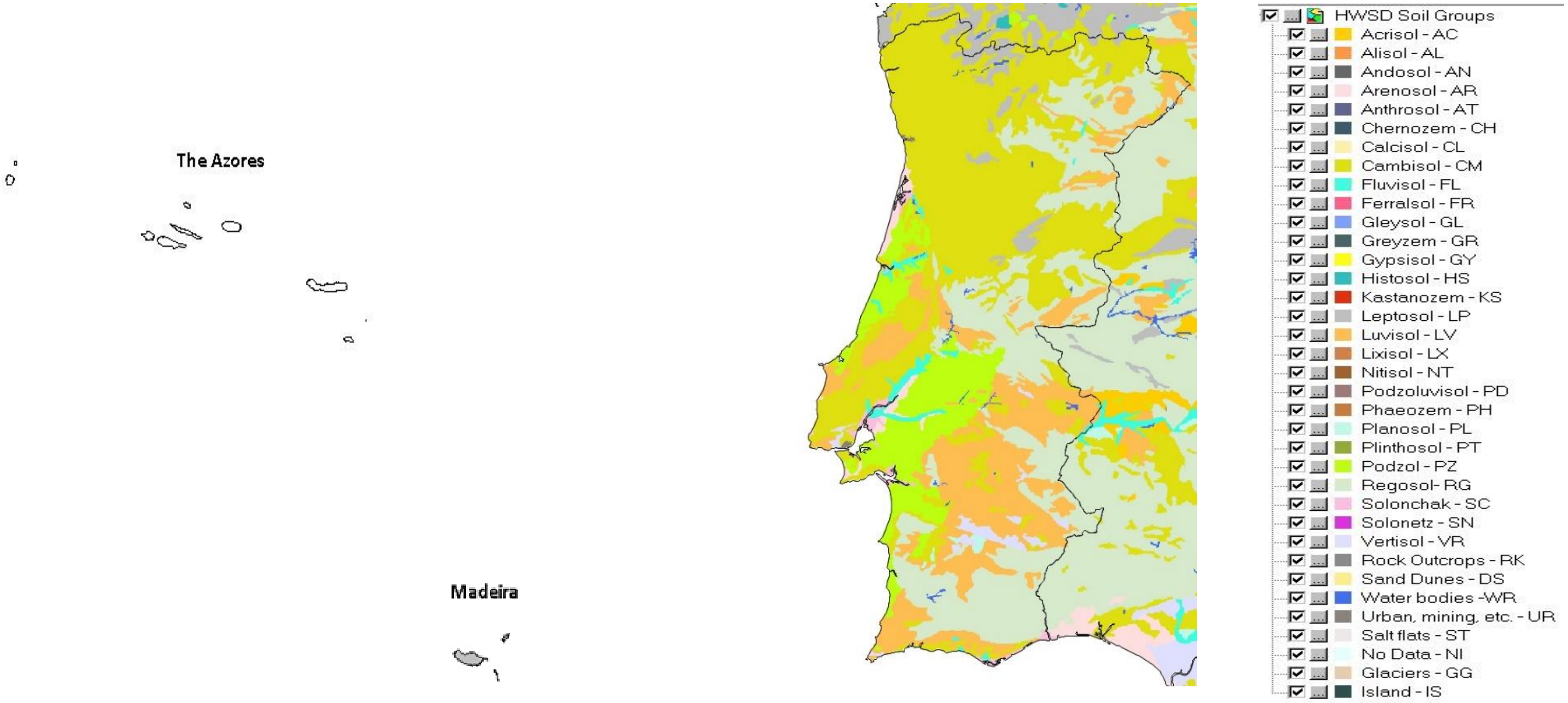

Supplementary Figure 4: Mainland Portugal and Portuguese autonomous regions soil map (adapted from FAO/IIASA/ISRIC/ISS-CAS/JRC, 2012. Harmonized World Soil Database (version 1.21). FAO, Rome, Italy and IIASA, Laxenburg, Austria). No data is available for The Azores.
